# Circulating metabolome landscape in Lynch syndrome

**DOI:** 10.1186/s40170-024-00331-9

**Published:** 2024-02-05

**Authors:** Tiina A. Jokela, Jari E. Karppinen, Minta Kärkkäinen, Jukka-Pekka Mecklin, Simon Walker, Toni T. Seppälä, Eija K. Laakkonen

**Affiliations:** 1 Gerontology Research Center and Faculty of Sport and Health Sciences, University of Jyväskylä, Jyväskylä, Finland; 2 Department of Surgery, The Wellbeing Services County of Central Finland, Jyväskylä, Finland; 3 Faculty of Sport and Health Sciences, University of Jyväskylä, Jyväskylä, Finland; 4 Department of Clinical Medicine, Faculty of Medicine and Health Technology, University of Tampere, Tampere, Finland; 5 Applied Tumor Genomics Research Program, Research Programs Unit, University of Helsinki, Helsinki, Finland; 6 Department of Abdominal Surgery, Helsinki University Hospital and University of Helsinki, Helsinki, Finland; 7 Department of Gastroenterology and Alimentary Tract Surgery and TAYS Cancer Centre, Tampere University Hospital, Tampere, Finland; 8 Obesity Research Unit, Research Program for Clinical and Molecular Metabolism, Faculty of Medicine, University of Helsinki, Helsinki, Finland

**Keywords:** Metabolomic biomarkers, DNA mismatch repair deficiency, Hereditary cancer, Lipid metabolism, Cholesterol metabolism, Circulating amino acids, Ketone bodies, GlycA

## Abstract

Circulating metabolites systemically reflect cellular processes and can modulate the tissue microenvironment in complex ways, potentially impacting cancer initiation processes. Genetic background increases cancer risk in individuals with Lynch syndrome; however, not all carriers develop cancer. Various lifestyle factors can influence Lynch syndrome cancer risk, and lifestyle choices actively shape systemic metabolism, with circulating metabolites potentially serving as the mechanical link between lifestyle and cancer risk. This study aims to characterize the circulating metabolome of Lynch syndrome carriers, shedding light on the energy metabolism status in this cancer predisposition syndrome.

This study consists of a three-group cross-sectional analysis to compare the circulating metabolome of cancer-free Lynch syndrome carriers, sporadic colorectal cancer (CRC) patients, and healthy non-carrier controls. We detected elevated levels of circulating cholesterol, lipids, and lipoproteins in LS carriers. Furthermore, we unveiled that Lynch syndrome carriers and CRC patients displayed similar alterations compared to healthy non-carriers in circulating amino acid and ketone body profiles. Overall, cancer-free Lynch syndrome carriers showed a unique circulating metabolome landscape.

This study provides valuable insights into the systemic metabolic landscape of Lynch syndrome individuals. The findings hint at shared metabolic patterns between cancer-free Lynch syndrome carriers and CRC patients.

**Supplementary Information:**

The online version contains supplementary material available at 10.1186/s40170-024-00331-9.

## Background

Lynch syndrome (LS) is a hereditary condition caused by specific pathogenic mutations in DNA mismatch repair (*MMR*) genes, including *MLH1*, *MSH2*, *MSH6*, or *PMS2*. These mutations impair the cells’ ability to correct errors that occur during DNA replication. Individuals with LS face a significantly increased lifetime risk of developing cancers, with up to a 16-fold higher risk depending on the specific *MMR* gene affected [1, 2]. Colorectal cancer (CRC) is the most common cancer with a 52–97% lifetime risk when mutations occur in the *MLH1* and *MSH2* genes, 13–19% with mutated *MSH6* gene, and 10% with mutated *PMS2* gene [1, 2]. However, it is worth noting that not all individuals with LS develop cancer. The fact that some LS carriers remain cancer-free throughout their lives shows that cancer risk can be modified. Lifestyle factors, such as engaging in regular physical activity and maintaining a healthy body weight, are associated with a reduced cancer risk within the LS population [3].

The circulating metabolome reflects whole-body metabolic processes, which are influenced by genes, lifestyle factors, and health status [4–7]. Based on findings that adiposity-linked circulating metabolite signature is associated with elevated CRC risk [8], while a metabolite profile reflecting a healthy lifestyle is associated with lower CRC risk [5, 9] circulating metabolome holds the potential for characterizing a phenotype susceptible to CRC development.

Compelling evidence suggests that some circulating metabolites are causally related to cancer development. Lipids and amino acids were the most abundant circulating metabolites associated with CRC risk [5, 8–10]. Elevated levels of triglycerides, phospholipids, and cholesterol may promote cancer cell growth and proliferation by serving as an energy source and inhibiting CD8 + T cell proliferation [11]. Amino acids function as building blocks of proteins, precursors of various signaling molecules, and energy sources. Levels of certain amino acids, such as Alanine and Histidine, have been shown to inversely associate with the cancer stage [10]. In addition, Histidine concentration in blood was shown to be inversely associated with CRC risk [10]. Furthermore, circulating amino acid levels can influence immune cell activity, potentially impacting cancer development, as amino acids are vital for the basal metabolism of immune cells, and activated immune cells require more amino acids [12]. Collectively, these findings suggest that changes in circulating metabolite levels can precede CRC development. However, it remains unexplored whether the LS genotype affects the circulating metabolome. Therefore, our study investigated the circulating metabolome in cancer-free LS carriers.

In this study, we examined the circulating metabolome in a cohort of cancer-free LS carriers. We compared their metabolome to a control group of cancer-free non-carriers, as well as to a group of non-carriers with CRC. Our two main findings were that both LS and CRC participants exhibited similar patterns in circulating amino acids and ketone bodies. Second, we identified altered lipid metabolism in LS carriers compared with controls, which may play a role in the regulation of adiposity-related cancer risk. Overall, our study sheds light on the shared metabolic signatures of LS carriers, emphasizing the potential systemic factors at play in cancer susceptibility.

## Materials and methods

### Sample collection

Samples of three-group cross-sectional analysis were collected from different study cohorts; LS (*n* = 80), CRC (*n* = 89), and control (total *n* = 103)*.*

LS cohort included registered participants in the Finnish Lynch Syndrome Research Registry (LSRFi), with confirmed pathological *MMR* gene (*path_MMR*) variants (classes 4 and 5 by InSiGHT criteria) [13]. Sporadic CRC patients were enrolled at the time of their initial surgical appointment for CRC at the local tertiary center responsible for the management of CRC. Healthy non-carrier control samples were acquired from the Biobank of Eastern Finland (*n* = 76) and studies of the University of Jyväskylä (JYU) (*n* = 27) [4]. Informed consent was obtained from all participants, and ethical approval of sample collections was from: the Ethics committees of the Helsinki and Uusimaa Health Care District, Central Finland Health Care District the University of Jyväskylä. The study was conducted according to the guidelines of the Declaration of Helsinki.

All samples were taken in a fasted state. However, fasting instructions had slight differences. Control cohort participants fasted overnight and had no diet restrictions for the previous days. We do not have information about the length of the fasting of biobank samples. Samples of LS and CRC participants were taken after surveillance colonoscopy. According to colonoscopy protocol, LS and CRC participants were instructed to avoid eating high-fiber food (for example, fruit, berries, vegetables, and seeds) 2 days before the surveillance visit, to eat only easily digestible foods (for example yogurt, porridge, potato, pasta, fish, and white bread) a day before the surveillance visit and to abstain from solid food 12 h and any liquids 2 h before colonoscopy. From all participants, venous blood samples were taken from the antecubital vein to standard serum tubes. The samples were aliquoted and stored at – 80 °C until analysis.

### Metabolomics analysis

Metabolites were analyzed with a targeted proton nuclear magnetic resonance (^1^H-NMR) spectroscopy platform (Nightingale Health Ltd., Helsinki, Finland; biomarker quantification version 2020). In this high throughput ^1^H-NMR platform identification of small-molecule solutes present in native serum, including diverse amino acids and glycolysis substrates, is made through spectroscopic settings designed to minimize interference from the broad spectral signals emanating from lipoprotein particles. Additionally, the quantification of lipid constituents and assessment of the spectrum of fatty acid saturation levels was done using serum lipid extracts. The technical details of the method have been reported previously [14, 15]. The platform quantifies 250 metabolite measures. Of them, metabolome-wide analyses were conducted with 171 variables representing lipoproteins and lipids, glycolysis-related metabolites as well as amino acids, ketone bodies, and some other metabolites including GlycA, which is a measure of global N-acetyl glycosylation. Seventy-nine lipoprotein lipid ratios were omitted from the analyses as they mostly provide overlapping information with absolute lipid concentrations. For individual metabolite analyses, we concentrated on 65 key metabolites representing these metabolite groups.

### Statistical analysis

Descriptive statistics of each metabolite are reported in Supplement Table S1, and Table 1 shows the statistical analyses used in this study.

**Table 1:** The statistical analyses used in this study

Analysis	Data type	Software/package
Box-Cox data transformation was performed to ensure normally distributed data to follow up analysis. The Box-Cox transformation with lambda parameter estimated from data for each variable separately	Raw data	R version 4.0.0 or newer/MASS-package [16]
Principal coordinate analysis (PCoA) of the Euclidean distances calculated from circulating metabolite values	Box-Cox transformed data	R version 4.0.0 or newer/*ape* package [17]
PERMANOVA analysis was used to test whether cohorts’ centroids/mean in the PCoA distance matrix were significantly different from each other. Beta-dispersion (PERMDISP) test was used to examine whether the variance of cohorts was significantly different. ANOSIM test was used to determine whether there is more similarity within the cohorts than between cohorts	PcoA distance matrix	R version 4.0.0 or newer/*hagis* package [18]
Hierarchical clustering and heat mapping the Euclidean distance metric and the complete linkage method were used to create clusters based on similarity	The raw metabolite data was scaled column-wise to ensure that metabolite expression values were comparable across samples	R version 4.0.0 or newer/Pheatmap package [19]
Equality was tested using Levene’s test, and if at least one group showed heteroscedasticity, the ANCOVA test was replaced with a generalized linear model (GLiM)	Box-Cox transformed data	SPSS [20]
ANCOVA analysis, with covariates (age, sex, BMI), was used to evaluate whether the means of metabolite values were equal or not. The Sidak test was employed for multiple test correction during pairwise comparisons ANCOVA was performed on metabolites that had equal variances between groups	Box-Cox transformed data	SPSS [20]
A generalized linear model (GLiM) test, with covariates (age, sex, BMI), was used to evaluate whether the means of metabolites values are equal or not. The Sidak test was employed for multiple test correction during pairwise comparisons GLiM test was performed on metabolites that had non-equal variances between groups	Box-Cox transformed data	SPSS [20]
For data visualization, standardized mean differences (SMD) and SMD 95% confidence intervals were calculated and visualized in forest plot	Box-Cox transformed data	R version 4.0.0 or newer/MBESS-package [21], ggforestplot-package [22]

## Results

Descriptive characteristics of study subjects in LS carrier, control, and CRC cohorts are presented in Table 2.

**Table 2:** Descriptive characteristics of study subjects

Variable	LS	Control	CRC
*N* (total = 272)	80	103	89
Sex (*N*(%))			
Female	42(52.5%)	54 (52.4%)	39(43.8%)
Male	38(47.5%)	49 (47.6%)	50(56.2%)
Age, years (mean ± SD)	58.2 ± 13.3	59.7 ± 14.3	70.8 ± 9.6
Body mass index, kg/m^2^ (mean ± SD)	26.6 ± 5.5	27.6 ± 6.0	26.7 ± 4.9
*path_MMR* (*N*(%))			
*MLH1*	52(65%)	0	0
*MSH2*	13(16.25%)	0	0
*MSH6*	14 (17.5%)	0	0
*PMS2*	1(1.25%)	0	0

*LS path_MMR* carrier currently cancer-free, *Control* Non-carrier currently cancer-free, *CRC* Non-carrier colorectal cancer patient

### Circulating metabolome level results

#### Cancer-free LS carriers’ circulating metabolome profile showed more similarity with CRC patients’ profile than controls

**Fig. 1:**
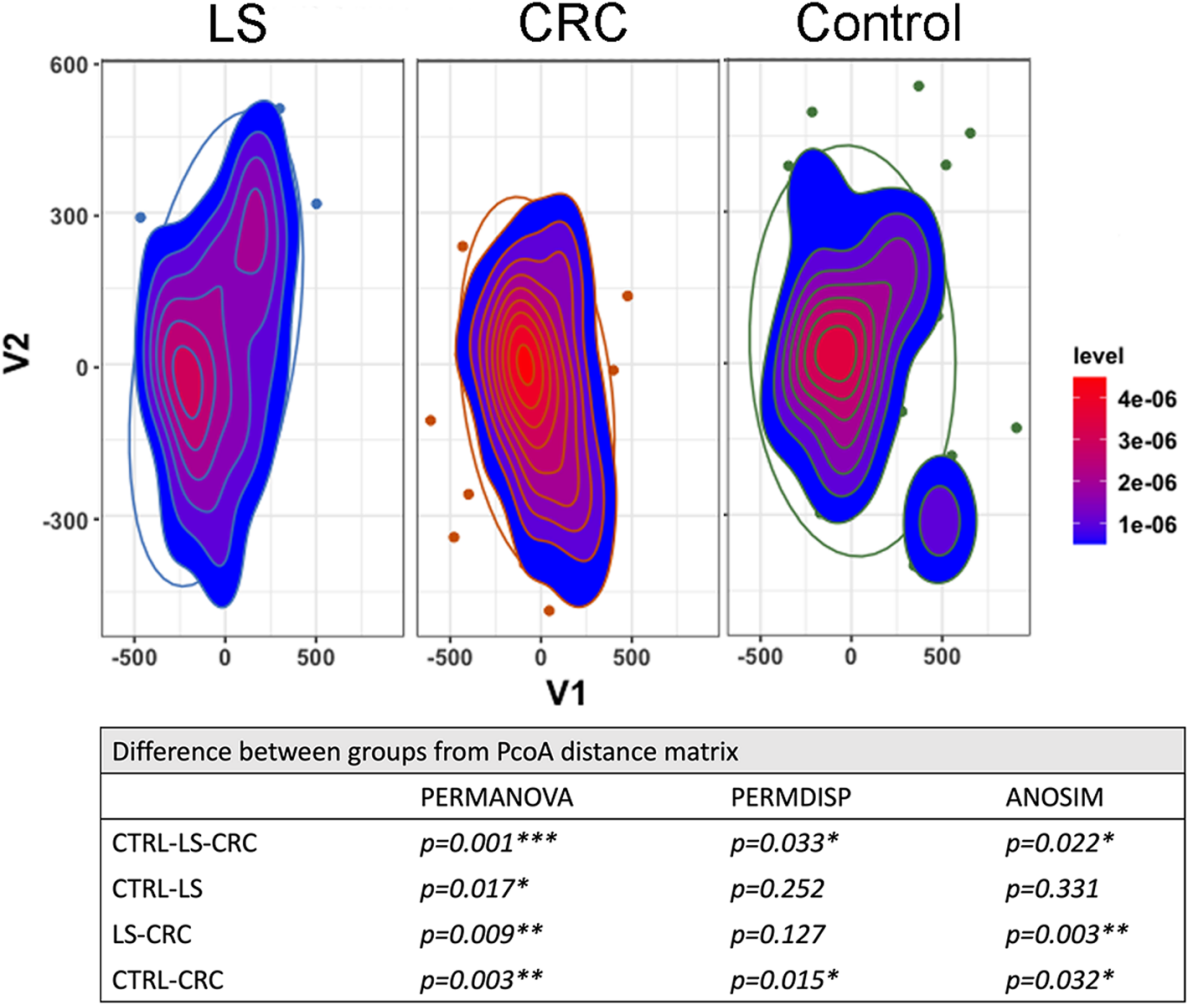
Principal coordinate analysis (PCoA) of the Euclidean distances calculated from 171 circulating metabolome values. After data dimension reduction the difference between cohorts), cancer-free Lynch syndrome carriers (LS), sporadic colorectal cancer patients (CRC) and cancer-free non-carrier controls (CTRL), was tested for significance using PERMANOVA on the PcoA distance matrices. PERMDISP test was used to test if the variance of cohorts was significantly different or not. ANOSIM test was used to test if there is more similarity within the cohorts than between cohorts compared to all cohorts and each cohort paired. The table shows p-values for PERMANOVA, PERMDISP, and ANOSIM analysis

One hundred seventy-one circulating metabolites were studied using NMR-based targeted analysis. The dimension reduction method PCoA and PERMANOVA test indicate that circulating metabolite profiles differed between the three groups (Fig. 1). Pairwise comparisons further showed that the metabolite profiles of all three groups were significantly different from each other (Fig. 1). In summary, cancer-free LS carriers have a significantly distinct circulating metabolome landscape when compared to healthy noncarrier control or CRC patient cohorts.

#### Path_MMR gene variants show some differences in circulating metabolome

Cancer risk in LS is strongly associated with *path_MMR* genes, where *MLH1* is the most aggressive gene to increase cancer risk [1, 2]. *MLH1* is also the primary mutation found in our Finnish LS cohort [23], which is why our path_MMR carrier groups are not equally sized (Table 2). These unbalanced group sizes need to be considered when interpreting the following results. Nevertheless, we considered it important to study whether different *path_MMR* genes have a different effect on circulating concentrations of the 171 metabolites and performed Euclidean clustering and heatmap visualization within the LS cohort (Supplement Figure S2). No apparent clustering was detected based on *path_MMR* genes (Supplement Figure S2). To study specific differences between groups carrying each of the *path_MMR* genes we excluded *PSM2,* since we only had one carrier in the cohort. When comparing *MLH1, MSH2*, and *MSH6* carrier groups PCoA and PERMANOVA showed that these three groups had some differences in circulating metabolome (*p* value = 0.032*). However, pairwise comparison did not show significant differences between different variants (Supplement Figure S1.). When comparing *MLH1* variant carrier group to the non-carrier cohort, we saw a significant difference, whereas *MSH6* variant carriers did not have a significant difference to the non-carrier control group. Conversely*, MLH1* carriers did not have a significant difference in the CRC patient cohort circulating metabolome in PERMANOVA analysis, and *MSH6* had a significant difference in the CRC cohort. In summary, these results suggest that *MLH1* carriers’ circulating metabolome is more similar with CRC patients and *MSH6* more similar with healthy non-carrier circulating metabolome.

### Circulating metabolites-specific results

#### Lipoprotein- and lipid-related alterations in LS and CRC cohorts compared to controls

**Fig. 2:**
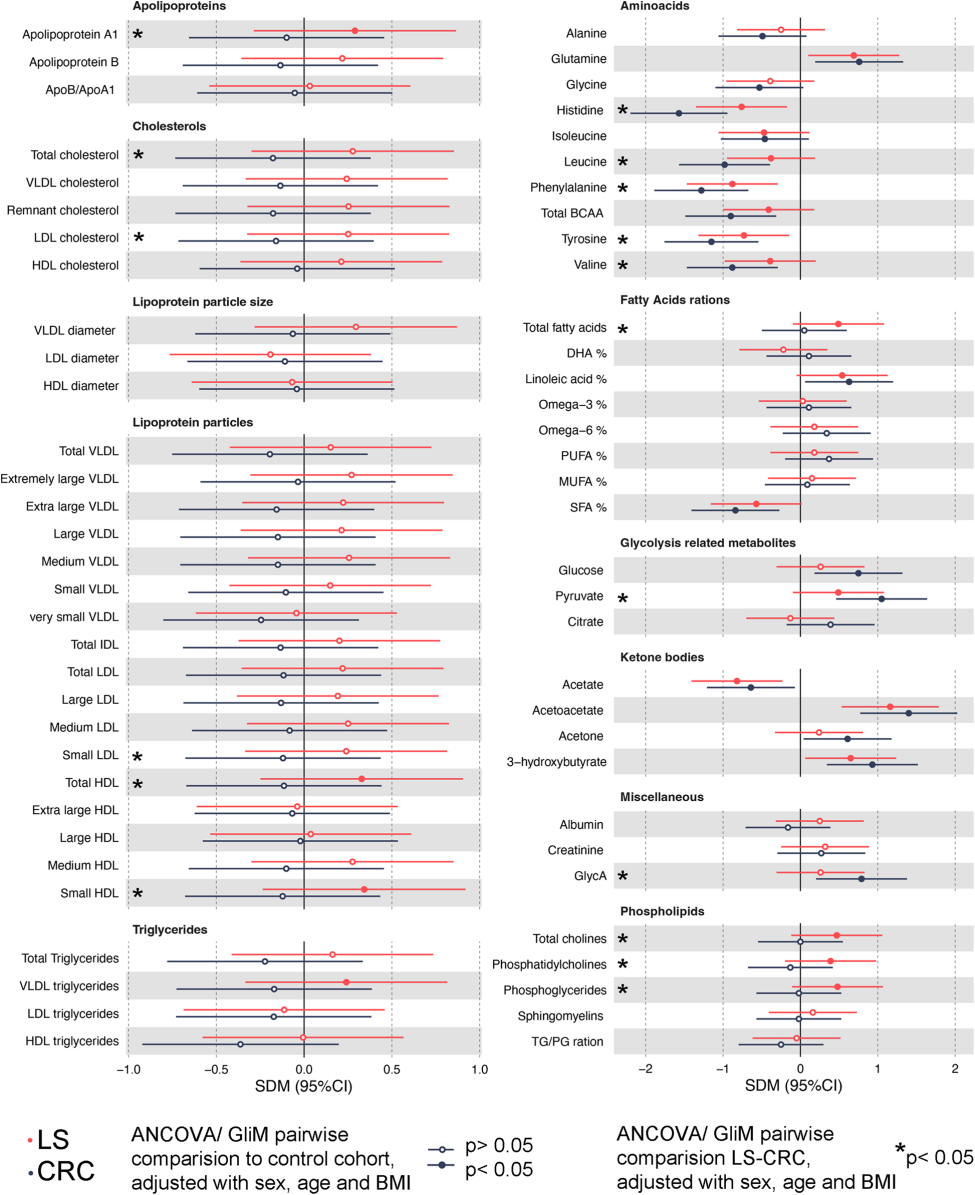
Forest plots illustrate standardized mean differences (SDM) relative to the control cohort, along with their corresponding 95% confidence intervals, calculated using box-cox transformed metabolite values. Significant differences between the control cohort and both LS and CRC cohorts are evaluated using ANCOVA or GLiM analysis, incorporating covariates age, sex, and BMI; a colored dot indicates corrected *p* value < 0.05 of statistical test compared to control cohort mean value, and a * indicates corrected *p* value < 0.05 in statistical test between LS and CRC cohort means. Test and values for all 65 key metabolites comparisons are shown in Supplementary Table S2

ANCOVA or GLiM analysis was employed, with covariates (age, sex, BMI), to examine 65 key metabolites (Fig. 2, Supplemental Table S2). Analyses revealed distinct metabolic alterations within the LS cohort compared to the control cohort, particularly in relation to lipoprotein particles and their lipid content (Fig. 2). The mean total cholesterol in the LS cohort and cholesterol bound to very low-density lipoprotein (VLDL), low-density lipoprotein (LDL), or high-density lipoprotein (HDL) particles were elevated in LS compared to control; however, these differences were not statistically significant after multiple test correction (Fig. 2).

Apolipoprotein A1 (ApoA1), a key constituent of HDL particles, displayed higher levels in the LS relative to the control or CRC cohort. Related to this, the LS cohort had higher amounts of total HDL particles but when particle sizes were inspected separately, only the amount of small-size HDL particles differed compared to other cohorts (Fig. 2). Furthermore, the LS compared to the control cohort exhibited heightened levels of triglycerides specifically localized within VLDL particles (Fig. 2). Elevated concentrations of total cholines, phosphatidylcholines, and phosphoglycerides were detected in the LS cohort when compared to the control and CRC group (Fig. 2). In contrast, the CRC cohort did not exhibit any significant alterations in lipoprotein and lipid metabolism-related metabolites when compared to the control cohort (Fig. 2).

#### Lipoprotein and lipid levels vary between different path_MMR carriers

**Fig. 3:**
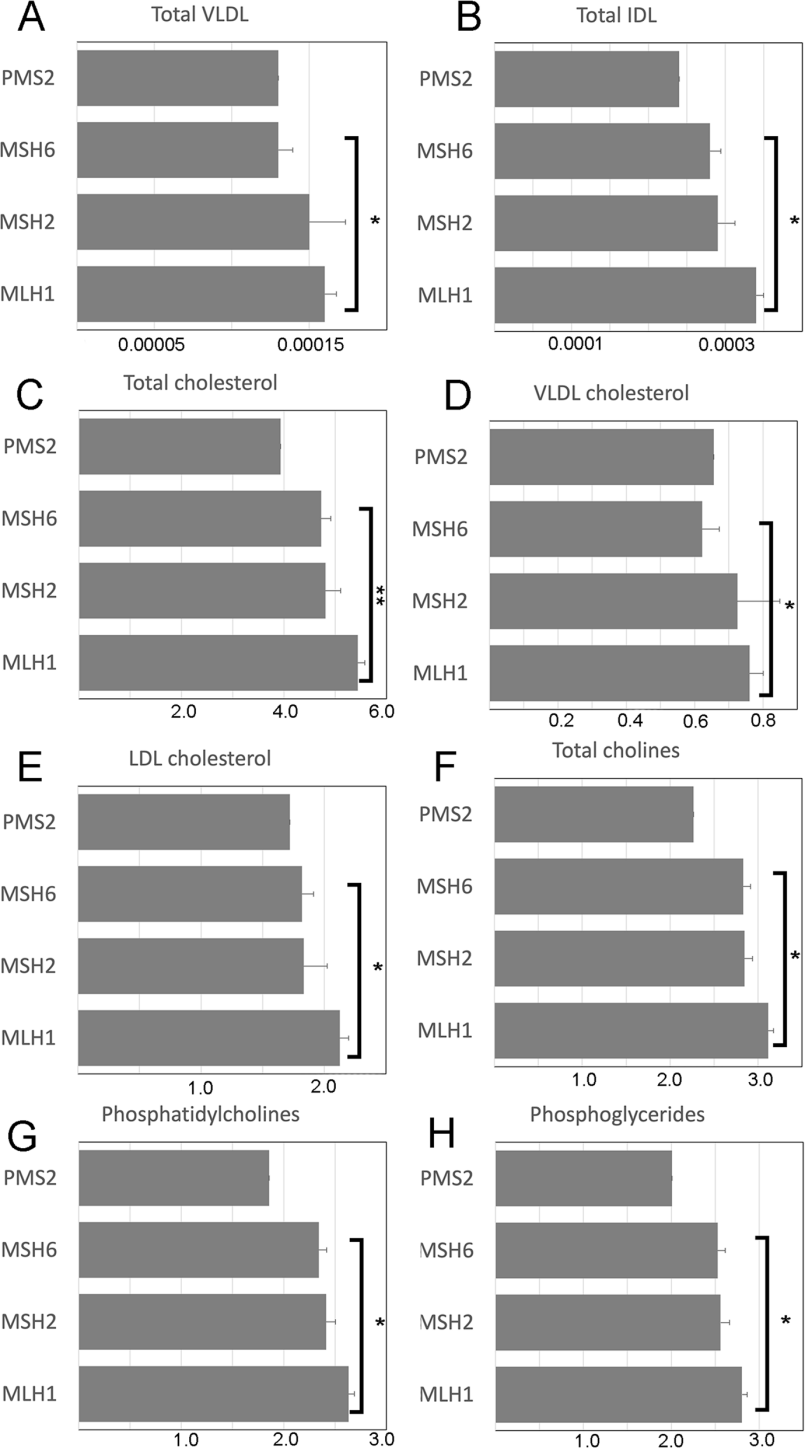
Metabolite levels in different *path_MMR* gene cohorts mmol/l (**A**–**H**). ANCOVA (**A**–**H**) test, incorporating covariates age, sex, and BMI, was used to test the difference between the *MLH1* cohort and other *path_MMR* cohorts, * = corrected *p* value < 0.05, ** = corrected *p* value < 0.01. Test and values for all 65 key metabolites comparisons are shown in Supplementary Table S3.

ANCOVA and GLiM analyses, with covariates (age, sex, BMI) were used to determine whether different *path_MMR* carriers express different levels of 65 selected non-redundant key metabolites (Supplemental Table S3). A finding was that MLH1 carriers had the highest circulating cholesterol levels (mean of total cholesterol, *MLH1* = 5.44 mmol/l, *MSH2* = 4.81 mmol/l, and *MSH6* = 4.72 mmol/l) and *MLH1* carriers had significantly higher cholesterol levels than *MSH6* carriers (Fig. 3C). Of the cholesterol transportation particles, the amounts of very low-density lipoprotein (VLDL) (Fig. 3A) and intermediate density lipoprotein (IDL) (Fig. 3B), were highest in *MLH1*-cohort, and significantly lower in *MSH6*-cohort when compared to *MLH1*-cohort (Fig. 3A, B). VLDL and LDL-bound cholesterol levels were also highest in the *MLH1* cohort (Fig. 3D, E). Additionally, phospholipids were upregulated in the *MLH1* cohort (Fig. 3F, G, H). In conclusion, the levels of most circulating metabolites exhibited similarity among different *path-MMR* carriers (Supplement Table S3). However, *MLH1* carriers demonstrated higher mean levels of lipoprotein and lipid-related metabolites when compared to *MSH6* carriers.

#### Circulating amino acids and ketone bodies show similarity between LS and CRC cohorts

In the LS and CRC cohort, glutamine levels were elevated, whereas all other studied amino acids; alanine, histidine, isoleucine, phenylalanine, tyrosine, valine, and total branched-chain amino acids (BCAAs) were curtailed compared to the control group (Fig. 2). GlycA levels were higher in LS and CRC cohorts when compared with the control group. However, after multiple test corrections, GlcyA levels in the LS cohort were not significantly higher (Fig. 2). When examining ketogenesis products, both CRC and LS cohorts had altered ketone body expression levels compared to the control cohort (Fig. 2). In summary, these results revealed that the LS cohort shows similarity with CRC cohort regarding circulating amino acids, ketone bodies, and inflammation marker GlycA signatures.

## Discussion

In this study, we investigated the circulating metabolome signature of 80 cancer-free carriers of LS and compared it to two distinct groups: a cancer-free non-carrier control cohort and a cohort of individuals with sporadic CRC. Our findings showed that the metabolomic signatures of LS carriers were unique and were statistically significantly different from noncarrier control and CRC cohort metabolomic signatures. No significant omics-level differences were found within LS carriers based on different *path_MMR* gene variants. However, our individual metabolite level inspections revealed notably higher phospholipids levels and other significant alterations related to lipoprotein—and lipid metabolism in LS carriers compared to control, which was not evident in the CRC-control comparison. Furthermore, we also identified within LS cohort differences; that *path_MLH1* carriers showed the highest levels of specific lipid and lipoprotein metabolite. Similar alterations in lipoprotein- and lipid metabolism were not detected in individuals with sporadic CRC. Additionally, both LS and CRC cohorts exhibited distinct yet parallel alterations in the circulating amino acids and ketone body levels.

The circulating metabolome is associated with cancer risk [5, 8, 9]. Germline mutations in DNA repair genes elevate cancer risk by imposing a high mutation load on fast-proliferating epithelial tissues. However, there is a limited understanding of the interaction between germline mutations in the DNA repair system and systemic metabolomics. DNA repair gene *BRCA1* has been shown to impact cellular metabolism [24, 25]. Additionally, women with this breast cancer predisposition gene exhibit an altered circulating metabolome signature [26]. In the context of CRC, *MLH1* deficiency in the CRC cell model has been found to disrupt mitochondrial metabolism [27]. Our findings revealed that LS carriers had a significantly altered circulating metabolome signature compared to the control cohort. Interestingly, this signature had some similarities to the circulating metabolome signature observed in sporadic CRC patients. These results, together with previous findings related to *BRCA1* and *path_MMR*, suggest that these cancer-predisposing germline mutations not only increase the mutation load in epithelial cells but also impact systemic metabolomic status.

The association between cancer risk and lipoprotein and lipid levels has been extensively studied, but the results remain controversial. A recent systemic meta-analysis showed that triglycerides and total cholesterol positively correlated with CRC incident rate, while high levels of HDL cholesterol negatively correlated with CRC incidences [28]. This analysis did not show an association between LDL cholesterol and CRC risk. However, some studies indicate a U-shaped association, suggesting that intermediate LDL cholesterol levels are related to the lowest cancer risk [29]. In the LS carriers with type two diabetes, triglyceride level was not, but cholesterol level was associated with higher CRC risk [30]. While there is no clear consensus on whether lipoprotein and lipid metabolism are associated with CRC risk or not, it is evident that lipoprotein and lipids play a critical role as functional molecules in various carcinogenesis-related processes. Dysregulation of lipid metabolism represents an important metabolic alteration in cancer. Lipoproteins and lipids act as energy producers, signaling molecules, and source material for the biogenesis of cell membranes [31]. Cholesterol is a key component of cell membrane lipid rafts, which play a vital role in cancer signaling. It can directly activate oncogenic signaling pathways [29, 32]. Moreover, cholesterol and lipoproteins are essential in triggering immune responses [32]. Our findings revealed that in comparison to the control cohort, cancer-free carriers of LS exhibited not significantly different but consistently higher cholesterol levels and alterations in the distribution of cholesterol-transporting lipoprotein particles. *MLH1* carriers with the highest cancer risk had the highest cholesterol levels. The elevated lipoprotein and lipid levels in LS carriers could be the response to the high levels of immune activity known to be present in LS. It is possible that increased lipoprotein and lipid levels support immune cell functions, aiding in the elimination of premalignant cells. On the other hand, elevated levels might also provide growth advantages to malignant cells by boosting oncogenic signaling and overall cell proliferation. The exact role of elevated lipoprotein and lipid levels in LS carcinogenesis, whether protective or oncogenic, remains to be thoroughly investigated in future studies.

Amino acids and ketone bodies have links to cancer progression. Amino acids are necessary building blocks for cancer cell protein synthesis, and the cancer cell ketone body’s metabolism has been shown to be disrupted [33, 34]. Interestingly, the circulating amino acid histidine has been found to have an inverse association with CRC risk [10]. Alterations in circulating amino acid levels have been reported in many cancers. A decrease in circulating amino acids is often suggested to be caused by cachexia. However, this may not be the sole reason, as similar decreases have been observed in cancer patients without weight loss or cachexia [34]. The reduction in amino acids could potentially be attributed to the high demand for amino acids by cancer cells [34]. Our results showed that LS carriers compared to the control group exhibited a similar decrease in circulating amino acids. This suggests that amino acids might be consumed by non-tumorous cells, for example, immune cells, in LS carriers.

Ketone bodies serve as an energy source and can be involved in various metabolic pathways. Produced in the liver, ketone bodies are transported to other tissues when needed. Due to impaired ketone metabolism in cancer cells, most cancer cells cannot utilize ketone bodies as an energy source, and ketone bodies can even impose reactive oxygen species production, inhibiting cancer cell growth [35]. Thus, ketone bodies possess anti-cancer properties [35]. However, the reasons behind LS carriers expressing a similar circulating ketone bodies’ profile as CRC patients remain unclear.

Inflammation and its biomarkers have been strongly associated with cancer risk, progression, and survival [36, 37]. GlycA, a novel inflammation marker has been linked to CRC incidence and mortality [38]. Elevated GlycA levels indicate both acute and chronic inflammation, serving as predictive markers for overall mortality. These levels remain persistently elevated and stable for an extended period, spanning up to a decade [39]. Our results showed increased GlycA levels in the CRC cohort, and in addition to that LS carriers displayed an increase in GlycA levels. This rise in GlycA level could be attributed to heightened immune activity in LS. However, within our current cohort size, the GlycA levels, when compared to the control cohort, did not reach statistical significance after multiple test corrections. Further investigation is warranted to determine if statistical significance can be observed with a larger LS cohort.

While our study provides valuable insights, it is important to acknowledge certain limitations. The sample size of our study, although comprehensive, might still be relatively small for detecting subtle differences in certain metabolomic parameters. A larger and more diverse cohort, including a greater representation of MSH2, MSH6, and PSM2 carriers, could potentially uncover more intricate details regarding how different path_MMR variants associate with distinct metabolic signatures. Additionally, it is essential to note that our LS and CRC cohorts were collected under different setups than control. LS and CRC serum samples were obtained prior to colonoscopy surveillance, whereas control samples originated from both biobank sources and University of Jyväskylä (JYU) studies. Although all samples were collected under an overnight fasted state, moderate variations in fasting instructions (detailed in the materials and methods section) could potentially impact metabolite levels. One example is the adherence to a low-fiber diet 2 days prior to colonoscopy. We are not aware of studies showing the effects of bowel preparation in serum metabolomics, thus, it is not clear whether this methodological issue would affect results. However, here we would like to raise the observed increase in ketone body levels in both LS carriers and CRC patients, which could be attributed to the fasting instructions they followed before blood sampling. LS and CRC cohorts had wider fasting guidelines as they underwent colonoscopy surveillance on the same day as blood collection, possibly leading to an upregulation of ketone bodies as a response to the energy deficit caused by fasting. However, beyond the fasting-related effects, it is also plausible that path_MMR genes influence overall cell ketone body metabolism, leading to alterations in the circulating ketone body levels. These genes might have broader implications for the metabolism of ketone bodies in the body. Additionally, it remains unclear whether there are specific systemic-level changes in ketone body consumption that could contribute to the observed increase in circulating ketone bodies in LS carriers and CRC patients. Further investigations are warranted to explore the potential interplay between path_MMR genes, systemic ketone body metabolism, and their implications in cancer development. In summary, while fasting-related factors might explain part of the increased ketone body levels in LS carriers and CRC patients, the role of path_MMR genes and systemic-level ketone body metabolism alterations deserves thorough investigation to gain a comprehensive understanding of their impact on cancer risk and metabolic processes. Furthermore, the lack of detailed lifestyle information for the different cohorts introduces an element of uncertainty, as lifestyle factors can significantly influence overall metabolite signatures. However, it is noteworthy that all cohorts consisted of Finnish individuals, and we have striven to balance the cohorts by sex and BMI to mitigate potential confounding effects. It is important to acknowledge that the mean age of the CRC patient cohort is higher than that of the LS cohort. This discrepancy arises from the fact that sporadic CRC incidences are most prevalent in older age groups, making it challenging to establish an exact age-matched Finnish LS cohort within the same age range. To address this, we incorporated age as a covariate in the ANCOVA analysis and expanded the control cohort to encompass participants across the full age spectrum represented by both the LS and CRC cohorts.

The cross-sectional design of our study limits our ability to infer causality or the temporal sequence of metabolic changes in relation to LS-related cancer development. Longitudinal studies tracking metabolic alterations over time would provide a more dynamic perspective on the interplay between systemic metabolism and cancer risk.

## Conclusions

The results demonstrate that the oncogenic stress imposed by *path_MMR* genes is reflected at the systemic metabolomic level. These constitutional alterations in energy metabolism may play a significant role in the etiology of LS-related cancers. The findings of our study raise several intriguing questions regarding the interaction between systemic metabolism and pre-carcinogenic processes.

### Supplementary Information

**Additional file 1: Supplement Figure S1.** Principal coordinate analysis (PCoA) of the Euclidean distances calculated from 171 circulating metabolome values. The difference between *path_MMR* variant carriers; *MLH1*, *MSH2* and *MSH6* was tested for significance using PERMANOVA on the PCoA distance matrices. Beta-dispersion test was used to test if the variance of cohorts was significantly different or not. ANOSIM test was used to test if there is more similarity within the cohorts than between cohorts A) compare all cohorts and B-D) each cohort paired. The table shows p-values for PERMANOVA, PERMDISP and ANOSIM analysis.

**Additional file 2: Supplement Figure S2.** Clustered heatmap based on Euclidean distance metric clustering and metabolite-wise scaled values. Different *path_MMR* gene variant carriers are presented in the right side color bar; *MLH1* = yellow, *MSH2* = blue, *MSH6* = green and *PMS2* = red

**Additional file 3: Supplementary Table S1.** Descriptive statistics for all 171 metabolites. **Supplementary Table S2.** ANCOVA& GLiM analysis for 65 key metabolites comparing LS, control and CRC cohorts. **Supplementary Table S3.** Descriptive statistics and ANCOVA& GLiM analysis for 65 key metabolites comparing different *path_MMR* gene carriers.

## Data Availability

The datasets used and analyzed during the current study are available from the corresponding author on reasonable request.

## Abbreviations

LS: Lynch syndrome
CRC: Colorectal cancer
MMR: DNA mismatch repair gene
path_MMR: Pathogenic DNA mismatch repair gene
GlycA: Global N-acetyl glycosylation
VLDL: Very low-density lipoprotein
IDL: Intermediate-density lipoprotein
LDL: Low-density lipoprotein
HDL: High-density lipoprotein
PCoA: Principal coordinate analysis
GLiM: Generalized linear model
