# Supplementary figures and images for: Circulating metabolome landscape in Lynch syndrome
Source: Cancer Metab. 2024 Feb 5;12:4. doi: 10.1186/s40170-024-00331-9 (PMC10840166; doi:10.1186/s40170-024-00331-9)

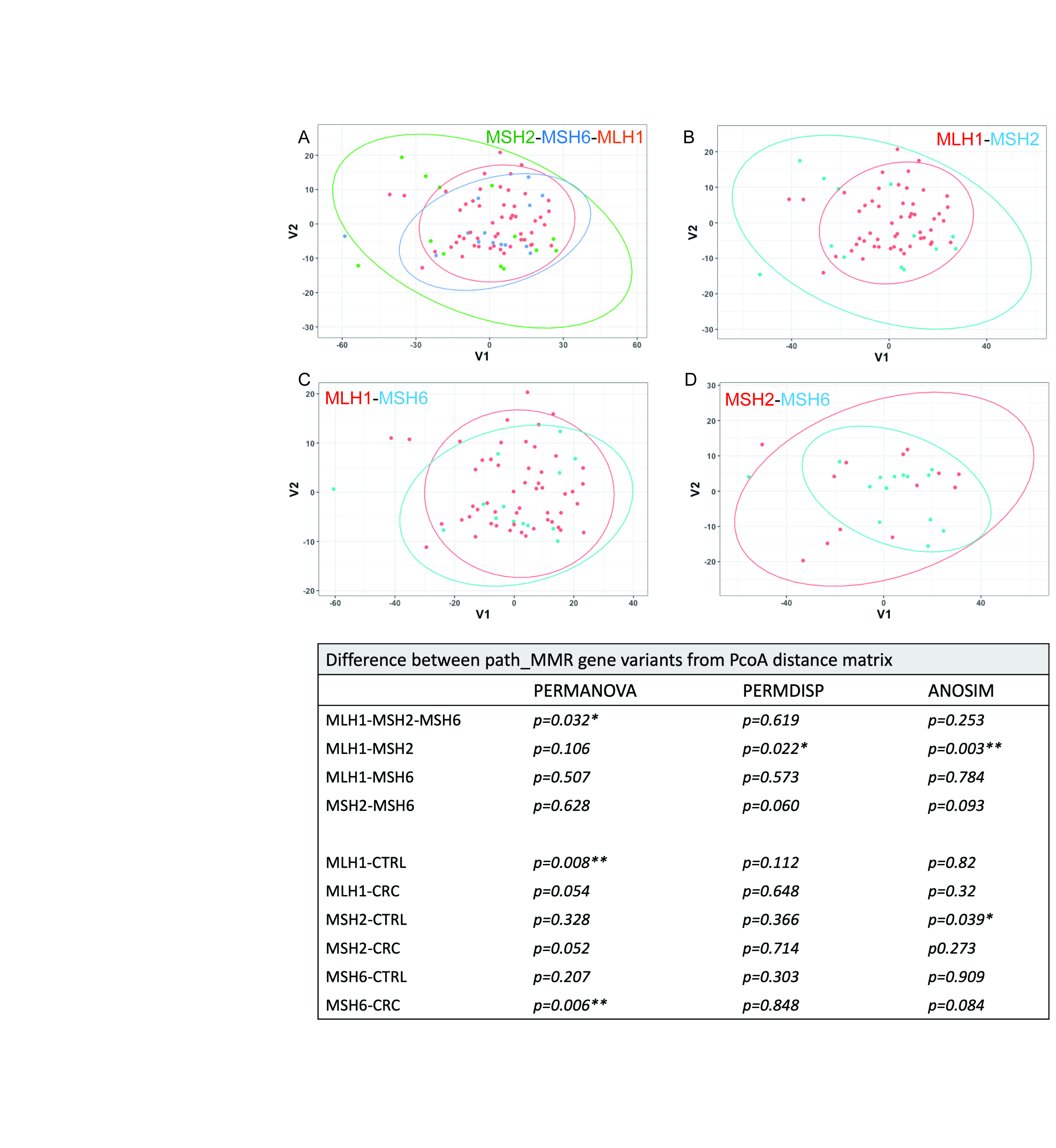

Supplement: Supplementary file 1 — Additional file 1: Supplement Figure S1. Principal coordinate analysis (PCoA) of the Euclidean distances calculated from 171 circulating metabolome values. The difference between path_MMR variant carriers; MLH1, MSH2 and MSH6 was tested for significance using PERMANOVA on the PCoA distance matrices. Beta-dispersion test was used to test if the variance of cohorts was significantly different or not. ANOSIM test was used to test if there is more similarity within the cohorts than between cohorts A) compare all cohorts and B-D) each cohort paired. The table shows p-values for PERMANOVA, PERMDISP and ANOSIM analysis. [file 40170_2024_331_MOESM1_ESM.tif]

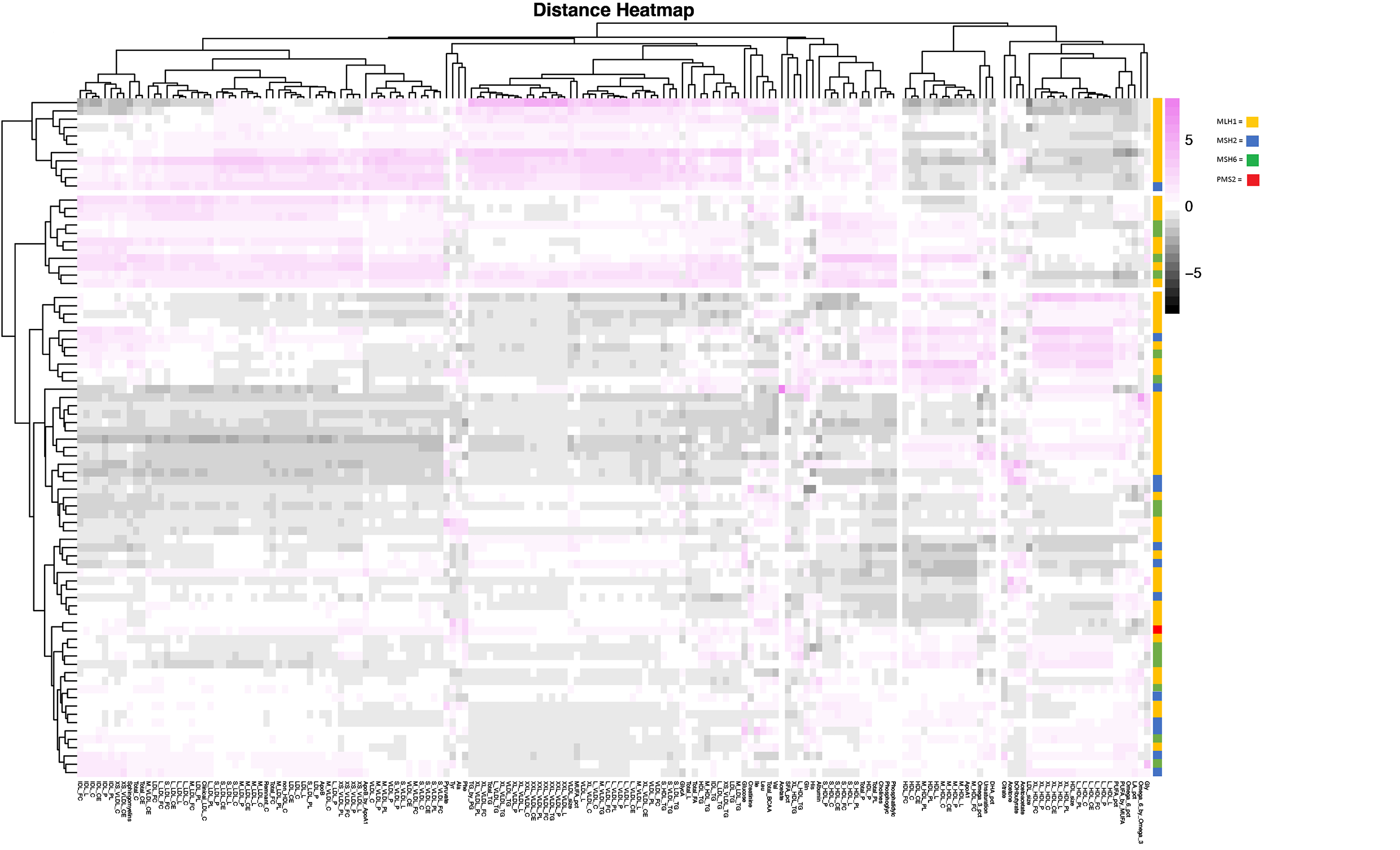

Supplement: Supplementary file 2 — Additional file 2: Supplement Figure S2. Clustered heatmap based on Euclidean distance metric clustering and metabolite-wise scaled values. Different path_MMR gene variant carriers are presented in the right side color bar; MLH1 = yellow, MSH2 = blue, MSH6 = green and PMS2 = red [file 40170_2024_331_MOESM2_ESM.tif]
