# Supplementary material for: Circulating metabolome landscape in Lynch syndrome
Source: Cancer Metab. 2024 Feb 5;12:4. doi: 10.1186/s40170-024-00331-9 (PMC10840166; doi:10.1186/s40170-024-00331-9)
Supplement: Supplementary file 3 — Additional file 3: Supplementary Table S1. Descriptive statistics for all 171 metabolites. Supplementary Table S2. ANCOVA& GLiM analysis for 65 key metabolites comparing LS, control and CRC cohorts. Supplementary Table S3. Descriptive statistics and ANCOVA& GLiM analysis for 65 key metabolites comparing different path_MMR gene carriers. [file 40170_2024_331_MOESM3_ESM.pdf]

Supplementary table 1

**Metabolite**	**Group**	**Unit**	**mean LS**	**mean CRC**	**mean CTRL**	**sd LS**	**sd CRC**	**sd CTRL**	**median LS**	**median CRC**	**median CTRL**	**IQR25% LS**	**IQR25% CRC**	**IQR25% CTRL**	**IQR75% LS**	**IQR75% CRC**	**IQR75% CTRL**
**Selected 65 key metabolites**																	
Apolipoprotein B	Apolipoproteins	g/l	0.9158232	0.8322972	0.8685833	0.2327816	0.2384698	0.2460206	0.8834791	0.7982708	0.8818804	0.7671389	0.6846178	0.6727900	1.0427727	0.9445055	1.0473977
Apolipoprotein A1	Apolipoproteins	g/l	1.6154785	1.5062682	1.5477829	0.2453775	0.2575796	0.3341829	1.6320978	1.4751815	1.5023638	1.4301000	1.3158421	1.3358334	1.7297629	1.6986119	1.7349222
Ratio of apolipoprotein B to apolipoprotein A1	Apolipoproteins	ratio	0.5810481	0.5612535	0.5782250	0.1795915	0.1652881	0.1880938	0.5624626	0.5324526	0.5450460	0.4602008	0.4548479	0.4351117	0.6704739	0.6376236	0.6825670
Total cholesterol	Cholesterol	mmol/l	5.1933437	4.6447197	4.8778695	1.0044610	1.1863552	1.3284805	5.1666296	4.5833250	4.8691620	4.2973890	3.8840820	3.8670031	5.8970836	5.4102492	5.8426317
VLDL cholesterol	Cholesterol	mmol/l	0.7293801	0.6194019	0.6591580	0.3022308	0.2540663	0.2596860	0.6442162	0.5730692	0.6431977	0.5560550	0.4376021	0.4564293	0.8703833	0.7487224	0.8571140
Remnant cholesterol (non-HDL, non-LDL -cholesterol)	Cholesterol	mmol/l	1.6314732	1.4328760	1.5240520	0.4478866	0.4841956	0.4941349	1.5463545	1.3887003	1.5202516	1.3135151	1.0860851	1.1313387	1.8680472	1.6465854	1.8867970
LDL cholesterol	Cholesterol	mmol/l	2.0189552	1.7605502	1.8757567	0.5402092	0.5663376	0.6871439	1.9739741	1.7458905	1.9339222	1.6521872	1.3945989	1.4141636	2.3050117	2.0985527	2.3781368
HDL cholesterol	Cholesterol	mmol/l	1.5429153	1.4512935	1.4780607	0.3566467	0.3658080	0.4249700	1.5364097	1.3714376	1.4146555	1.2730338	1.2077573	1.1399587	1.7060319	1.6778590	1.7619690
Average diameter for VLDL particles	Lipoprotein particle sizes	nm	38.8924	38.2643	38.4076	1.7014	1.2525	1.7033	38.7167	38.0999	38.2300	37.5651	37.5874	37.3252	39.6333	38.9568	39.5260
Average diameter for LDL particles	Lipoprotein particle sizes	nm	23.8915	23.9004	23.9110	0.0922	0.0836	0.1088	23.9067	23.9133	23.9153	23.8422	23.8599	23.8470	23.9556	23.9515	23.9794
Average diameter for HDL particles	Lipoprotein particle sizes	nm	9.6970	9.7024	9.7147	0.2452	0.2233	0.2577	9.6743	9.6618	9.6712	9.5362	9.5380	9.5263	9.8805	9.8479	9.8659
Concentration of VLDL particles	Lipoprotein particle conc	mmol/l	0.0001537	0.0001344	0.0001449	0.0000572	0.0000451	0.0000496	0.0001402	0.0001247	0.0001375	0.0001203	0.0001043	0.0001097	0.0001740	0.0001521	0.0001780
Concentration of chylomicrons and extremely large VLDL particles	Lipoprotein particle conc	mmol/l	0.0000016	0.0000011	0.0000008	0.0000023	0.0000019	0.0000009	0.0000007	0.0000005	0.0000004	0.0000001	0.0000000	0.0000000	0.0000021	0.0000014	0.0000013
Concentration of very large VLDL particles	Lipoprotein subclasses	mmol/l	0.0000043	0.0000028	0.0000035	0.0000039	0.0000019	0.0000034	0.0000030	0.0000024	0.0000026	0.0000017	0.0000015	0.0000014	0.0000049	0.0000042	0.0000048
Concentration of large VLDL particles	Lipoprotein subclasses	mmol/l	0.0000	0.0000	0.0000	0.0000	0.0000	0.0000	0.0000	0.0000	0.0000	0.0000	0.0000	0.0000	0.0000	0.0000	0.0000
Concentration of medium VLDL particles	Lipoprotein subclasses	mmol/l	0.0000	0.0000	0.0000	0.0000	0.0000	0.0000	0.0000	0.0000	0.0000	0.0000	0.0000	0.0000	0.0000	0.0000	0.0000
Concentration of small VLDL particles	Lipoprotein subclasses	mmol/l	0.0000	0.0000	0.0000	0.0000	0.0000	0.0000	0.0000	0.0000	0.0000	0.0000	0.0000	0.0000	0.0001	0.0000	0.0000
Concentration of very small VLDL particles	Lipoprotein subclasses	mmol/l	0.0001	0.0001	0.0001	0.0000	0.0000	0.0000	0.0001	0.0000	0.0001	0.0000	0.0000	0.0000	0.0001	0.0001	0.0001
Concentration of IDL particles	Lipoprotein subclasses	mmol/l	0.0003	0.0003	0.0003	0.0001	0.0001	0.0001	0.0003	0.0003	0.0003	0.0003	0.0002	0.0002	0.0004	0.0003	0.0004
Concentration of LDL particles	Lipoprotein particle conc	mmol/l	0.0013	0.0012	0.0012	0.0003	0.0003	0.0004	0.0013	0.0011	0.0013	0.0011	0.0010	0.0010	0.0015	0.0014	0.0015
Concentration of large LDL particles	Lipoprotein subclasses	mmol/l	0.0008	0.0007	0.0008	0.0002	0.0002	0.0002	0.0008	0.0007	0.0007	0.0007	0.0006	0.0006	0.0009	0.0008	0.0009
Concentration of medium LDL particles	Lipoprotein subclasses	mmol/l	0.0003	0.0003	0.0003	0.0001	0.0001	0.0001	0.0003	0.0003	0.0003	0.0003	0.0002	0.0002	0.0004	0.0003	0.0004
Concentration of small LDL particles	Lipoprotein subclasses	mmol/l	0.0002	0.0002	0.0002	0.0000	0.0000	0.0000	0.0002	0.0002	0.0002	0.0002	0.0001	0.0001	0.0002	0.0002	0.0002
Concentration of HDL particles	Lipoprotein particle conc	mmol/l	0.0172	0.0158	0.0162	0.0024	0.0027	0.0035	0.0173	0.0159	0.0160	0.0155	0.0138	0.0140	0.0183	0.0178	0.0187
Concentration of very large HDL particles	Lipoprotein subclasses	mmol/l	0.0003	0.0003	0.0003	0.0001	0.0001	0.0001	0.0002	0.0002	0.0002	0.0002	0.0002	0.0002	0.0003	0.0003	0.0003
Concentration of large HDL particles	Lipoprotein subclasses	mmol/l	0.0018	0.0017	0.0018	0.0009	0.0009	0.0010	0.0017	0.0015	0.0016	0.0011	0.0011	0.0010	0.0023	0.0022	0.0024
Concentration of medium HDL particles	Lipoprotein subclasses	mmol/l	0.0044	0.0040	0.0041	0.0009	0.0009	0.0012	0.0045	0.0039	0.0040	0.0037	0.0033	0.0034	0.0050	0.0047	0.0048
Concentration of small HDL particles	Lipoprotein subclasses	mmol/l	0.0107	0.0099	0.0100	0.0016	0.0015	0.0021	0.0104	0.0099	0.0103	0.0096	0.0089	0.0089	0.0117	0.0109	0.0114
Total triglycerides	Triglycerides	mmol/l	1.5447	1.1947	1.3915	0.9150	0.4630	0.8161	1.2568	1.1013	1.1711	0.9400	0.8610	0.8981	1.7269	1.5342	1.6317
Triglycerides in VLDL	Triglycerides	mmol/l	1.1318	0.8022	0.9666	0.8251	0.3958	0.7408	0.8509	0.6944	0.7543	0.5765	0.5206	0.5217	1.2426	1.1384	1.2168
Triglycerides in LDL	Triglycerides	mmol/l	0.1652	0.1618	0.1712	0.0421	0.0394	0.0480	0.1605	0.1557	0.1601	0.1371	0.1384	0.1380	0.1873	0.1768	0.1985
Triglycerides in HDL	Triglycerides	mmol/l	0.1410	0.1234	0.1403	0.0505	0.0410	0.0524	0.1304	0.1209	0.1307	0.1038	0.0971	0.1070	0.1722	0.1408	0.1578
Alanine	Amino acids	mmol/l	0.4359	0.4151	0.4630	0.0838	0.0893	0.1048	0.4193	0.3979	0.4374	0.3749	0.3630	0.3789	0.4974	0.4592	0.5378
Glutamine	Amino acids	mmol/l	0.7627	0.7659	0.6930	0.0985	0.0868	0.1028	0.7579	0.7649	0.6915	0.7017	0.7094	0.6199	0.8159	0.8178	0.7724
Glycine	Amino acids	mmol/l	0.2902	0.2777	0.3182	0.0898	0.0786	0.0901	0.2624	0.2612	0.3017	0.2307	0.2342	0.2530	0.3185	0.3094	0.3603
Histidine	Amino acids	mmol/l	0.0798	0.0697	0.0889	0.0098	0.0111	0.0132	0.0812	0.0699	0.0880	0.0735	0.0630	0.0808	0.0858	0.0764	0.0970
Isoleucine	Amino acids	mmol/l	0.0576	0.0574	0.0668	0.0171	0.0156	0.0246	0.0555	0.0551	0.0618	0.0469	0.0450	0.0510	0.0647	0.0651	0.0779
Leucine	Amino acids	mmol/l	0.1263	0.1063	0.1421	0.0309	0.0293	0.0464	0.1237	0.1019	0.1312	0.1068	0.0853	0.1129	0.1472	0.1242	0.1647
Phenylalanine	Amino acids	mmol/l	0.0705	0.0649	0.0886	0.0115	0.0122	0.0245	0.0686	0.0633	0.0858	0.0633	0.0560	0.0703	0.0761	0.0694	0.1032
Total concentration of branched-chain amino acids (BCAA, leucine + isoleucine + valine)	Amino acids	mmol/l	0.4274	0.3811	0.4732	0.0912	0.0906	0.1270	0.4242	0.3691	0.4479	0.3664	0.3183	0.3897	0.4874	0.4291	0.5256
Tyrosine	Amino acids	mmol/l	0.0670	0.0603	0.0800	0.0139	0.0161	0.0214	0.0659	0.0578	0.0755	0.0560	0.0483	0.0659	0.0748	0.0680	0.0896
Valine	Amino acids	mmol/l	0.2425	0.2173	0.2643	0.0471	0.0508	0.0619	0.2429	0.2128	0.2561	0.2049	0.1830	0.2233	0.2744	0.2417	0.2803
Total fatty acids	Fatty acids	mmol/l	14.1821	12.7105	12.8180	3.1814	2.3211	3.3581	13.2671	12.3330	13.1192	12.1692	11.2043	10.4888	15.3761	13.7452	14.2085
Ratio of docosahexaenoic acid (DHA) to total fatty acids	Fatty acids	%	2.3136	2.5077	2.4533	0.5210	0.5804	0.6387	2.2940	2.4106	2.3853	1.9296	2.0673	2.0707	2.6247	2.8344	2.8053
Ratio of linoleic acid to total fatty acids	Fatty acids	%	31.4571	31.7259	28.8886	4.0791	3.1127	5.0210	31.6402	31.6352	29.9561	28.2075	29.5707	25.2597	34.9088	33.8577	32.5758
Ratio of omega-3 fatty acids to total fatty acids	Fatty acids	%	4.9404	5.0549	4.9229	1.2264	1.3106	1.4009	4.8341	4.9904	4.9117	4.3569	4.1689	3.8929	5.5668	5.7714	5.5782
Ratio of omega-6 fatty acids to total fatty acids	Fatty acids	%	36.5190	37.0886	35.8021	3.9518	2.8668	4.1382	37.2534	37.2417	37.1383	33.4244	35.6389	33.9612	39.6794	38.5928	38.6641
Ratio of polyunsaturated fatty acids (PUFA) to total fatty acids	Fatty acids	%	41.4594	42.1435	40.7250	3.9939	2.9442	4.2449	42.4149	42.2518	41.6266	38.9449	40.0499	38.2326	44.1927	44.4732	43.6575
Ratio of monounsaturated fatty acids (MUFA) to total fatty acids	Fatty acids	%	25.2237	24.9741	24.7739	3.0922	2.5165	2.7614	24.7491	25.1081	24.3462	23.1671	22.9608	22.7909	26.6891	26.7341	26.5313
Ratio of saturated fatty acids (SFA) to total fatty acids	Fatty acids	%	33.3169	32.8824	34.5011	1.8570	1.3659	2.2537	32.9479	32.8806	34.5574	31.9542	31.9309	32.9530	34.6064	33.7818	35.7642
Glucose	Glycolysis related metab	mmol/l	5.9153	6.8700	5.7797	1.0796	1.8227	1.6302	5.6306	6.2303	5.4130	5.2856	5.6633	4.8677	6.3313	7.8359	6.3438
Pyruvate	Glycolysis related metab	mmol/l	0.0722	0.0885	0.0607	0.0330	0.0295	0.0380	0.0635	0.0866	0.0504	0.0485	0.0653	0.0356	0.0844	0.1054	0.0718
Citrate	Glycolysis related metab	mmol/l	0.0684	0.0762	0.0705	0.0093	0.0150	0.0126	0.0677	0.0759	0.0687	0.0605	0.0658	0.0618	0.0746	0.0847	0.0777
Acetate	Ketone bodies	mmol/l	0.0704	0.1011	0.0277	0.0586	0.0978	0.0355	0.0512	0.0698	0.0168	0.0279	0.0315	0.0100	0.0932	0.1310	0.0261
Acetoacetate	Ketone bodies	mmol/l	0.0285	0.0386	0.0247	0.0177	0.0318	0.0146	0.0242	0.0256	0.0205	0.0173	0.0203	0.0159	0.0340	0.0443	0.0281
Acetone	Ketone bodies	mmol/l	81.1801	80.9345	75.9279	17.5573	18.9598	13.8633	78.4399	78.5487	75.5640	68.7034	67.7710	65.8063	90.1157	90.2899	86.0268
3-Hydroxybutyrate	Ketone bodies	mmol/l	0.0290	0.0211	0.0277	0.0674	0.0109	0.0140	0.0153	0.0183	0.0239	0.0121	0.0137	0.0186	0.0201	0.0258	0.0310
Albumin	Fluid balance	g/l	0.8935	0.9845	0.8550	0.1493	0.1767	0.1545	0.8889	0.9666	0.8316	0.7967	0.8737	0.7531	0.9986	1.0660	0.9414
Creatinine	Fluid balance	µmol/l	41.7745	39.4910	40.1751	4.1818	5.2627	6.5182	42.3972	40.6092	41.6317	38.9482	36.4766	38.4916	45.1891	43.4873	43.7859
Glycoprotein acetyls	Inflammation	mmol/l	0.0000	0.0000	0.0000	0.0000	0.0000	0.0000	0.0000	0.0000	0.0000	0.0000	0.0000	0.0000	0.0000	0.0000	0.0000
Total cholines	Other lipids	mmol/l	3.0110	2.7634	2.7767	0.4260	0.4714	0.6084	3.0416	2.7187	2.7650	2.6262	2.4761	2.3264	3.2970	3.0364	3.1840
Phosphatidylcholines	Other lipids	mmol/l	2.5358	2.2833	2.3684	0.3968	0.4168	0.5677	2.5646	2.2295	2.3347	2.2225	2.0241	1.9726	2.7758	2.5556	2.6980
Phosphoglycerides	Other lipids	mmol/l	2.7073	2.4552	2.4808	0.4213	0.4410	0.5846	2.7431	2.4150	2.4778	2.3504	2.1699	2.0795	2.9787	2.7225	2.8359

Sphingomyelins	Other lipids	mmol/l	0.5086	0.4937	0.4957	0.0764	0.0928	0.0961	0.5053	0.4810	0.4902	0.4496	0.4358	0.4321	0.5619	0.5476	0.5581
Ratio of triglycerides to phosphoglycerides	Other lipids	ratio	0.5609	0.4927	0.5636	0.2919	0.1801	0.2706	0.4843	0.4734	0.5202	0.3583	0.3641	0.3598	0.6713	0.6356	0.7162
**All other metabolite variables**																	
Clinical LDL cholesterol	Cholesterol	mmol/l	2.9039	2.5447	2.6956	0.8320	0.9253	1.0602	2.8620	2.5147	2.7368	2.3273	1.9583	1.9793	3.3821	3.0940	3.5200
Total cholesterol minus HDL-C	Cholesterol	mmol/l	3.6504	3.1934	3.3998	0.9752	1.0401	1.1649	3.5551	3.1415	3.4621	2.9630	2.4158	2.5455	4.1907	3.7350	4.2598
Cholesteryl esters in HDL	Cholesteryl esters	mmol/l	1.2026	1.1341	1.1457	0.2782	0.2841	0.3310	1.2008	1.0566	1.1010	0.9960	0.9509	0.8703	1.3273	1.3026	1.3677
Cholesteryl esters in LDL	Cholesteryl esters	mmol/l	1.4843	1.2831	1.3719	0.4131	0.4147	0.5086	1.4448	1.2496	1.4192	1.2105	1.0118	1.0241	1.6854	1.5195	1.7433
Cholesteryl esters in VLDL	Cholesteryl esters	mmol/l	0.4245	0.3727	0.3890	0.1569	0.1538	0.1458	0.3812	0.3447	0.3868	0.3287	0.2651	0.2772	0.5255	0.4494	0.5122
Total esterified cholesterol	Cholesteryl esters	mmol/l	3.7924	3.3928	3.5568	0.7186	0.8577	0.9728	3.7954	3.3569	3.6174	3.1659	2.8407	2.8656	4.3010	3.9419	4.3027
Degree of unsaturation	Fatty acids	degree	1.3329	1.3358	1.3044	0.0751	0.0682	0.0853	1.3382	1.3264	1.3058	1.2775	1.2795	1.2544	1.3745	1.3892	1.3634
Ratio of omega-6 fatty acids to omega-3 fatty acids	Fatty acids	ratio	7.9937	7.8727	7.8656	3.0438	2.3133	2.4876	7.4396	7.4488	7.4276	6.2729	6.4470	5.9816	8.7303	8.8718	9.0877
Ratio of polyunsaturated fatty acids to monounsaturated fatty acids	Fatty acids	ratio	1.6825	1.7153	1.6795	0.3344	0.2872	0.3330	1.7175	1.6935	1.6964	1.4717	1.4888	1.4707	1.8813	1.9365	1.9170
Free cholesterol in HDL	Free cholesterol	mmol/l	0.3403	0.3172	0.3323	0.0806	0.0838	0.0967	0.3394	0.3009	0.3096	0.2800	0.2604	0.2694	0.3797	0.3680	0.3959
Free cholesterol in LDL	Free cholesterol	mmol/l	0.5346	0.4774	0.5038	0.1350	0.1544	0.1823	0.5283	0.4589	0.5142	0.4330	0.3783	0.3785	0.6099	0.5668	0.6388
Free cholesterol in VLDL	Free cholesterol	mmol/l	0.3049	0.2467	0.2701	0.1507	0.1031	0.1213	0.2561	0.2233	0.2519	0.2148	0.1689	0.1772	0.3454	0.2904	0.3509
Total free cholesterol	Free cholesterol	mmol/l	1.4009	1.2520	1.3211	0.2931	0.3323	0.3605	1.3461	1.2365	1.3464	1.1866	1.0330	1.0417	1.6009	1.4387	1.5729
Glycerol	Glycolysis related metab	mmol/l	0.1571	0.2608	0.0796	0.1664	0.2943	0.1067	0.1020	0.1513	0.0397	0.0411	0.0610	0.0235	0.2073	0.3456	0.0775
Total concentration of lipoprotein particles	Lipoprotein particle conc	mmol/l	0.0190	0.0175	0.0179	0.0025	0.0029	0.0037	0.0191	0.0176	0.0175	0.0170	0.0154	0.0157	0.0201	0.0195	0.0208
Cholesterol in chylomicrons and extremely large VLDL	Lipoprotein subclasses	mmol/l	0.0545	0.0345	0.0350	0.0606	0.0255	0.0408	0.0335	0.0270	0.0251	0.0182	0.0162	0.0072	0.0695	0.0538	0.0442
Cholesterol in IDL	Lipoprotein subclasses	mmol/l	0.9021	0.8135	0.8649	0.2205	0.2635	0.2848	0.8973	0.7824	0.8354	0.7341	0.6069	0.6759	1.0494	0.9675	1.0748
Cholesterol in large HDL	Lipoprotein subclasses	mmol/l	0.3796	0.3685	0.3742	0.2080	0.1900	0.2160	0.3517	0.3353	0.3370	0.2341	0.2360	0.2107	0.4873	0.4775	0.5032
Cholesterol in large LDL	Lipoprotein subclasses	mmol/l	1.3003	1.1366	1.2203	0.3354	0.3731	0.4485	1.2652	1.1395	1.2463	1.0560	0.9034	0.9120	1.4718	1.3498	1.5380
Cholesterol in large VLDL	Lipoprotein subclasses	mmol/l	0.1073	0.0812	0.0916	0.0745	0.0451	0.0596	0.0877	0.0730	0.0820	0.0593	0.0489	0.0500	0.1249	0.0986	0.1349
Cholesterol in medium HDL	Lipoprotein subclasses	mmol/l	0.5719	0.5282	0.5411	0.1260	0.1289	0.1601	0.5768	0.5163	0.5217	0.4816	0.4358	0.4091	0.6596	0.6192	0.6250
Cholesterol in medium LDL	Lipoprotein subclasses	mmol/l	0.5081	0.4363	0.4607	0.1606	0.1445	0.1824	0.4960	0.4191	0.4748	0.4023	0.3411	0.3393	0.5802	0.5227	0.5987
Cholesterol in medium VLDL	Lipoprotein subclasses	mmol/l	0.1775	0.1517	0.1619	0.0696	0.0766	0.0751	0.1659	0.1451	0.1613	0.1284	0.1013	0.0991	0.2177	0.1960	0.2183
Cholesterol in small HDL	Lipoprotein subclasses	mmol/l	0.4976	0.4598	0.4671	0.0723	0.0698	0.0948	0.4901	0.4612	0.4811	0.4416	0.4112	0.4122	0.5449	0.5105	0.5276
Cholesterol in small LDL	Lipoprotein subclasses	mmol/l	0.2106	0.1877	0.1948	0.0569	0.0531	0.0628	0.2056	0.1850	0.1987	0.1721	0.1527	0.1501	0.2327	0.2160	0.2433
Cholesterol in small VLDL	Lipoprotein subclasses	mmol/l	0.1595	0.1474	0.1497	0.0571	0.0581	0.0535	0.1495	0.1362	0.1461	0.1244	0.1081	0.1083	0.1873	0.1726	0.1879
Cholesterol in very large HDL	Lipoprotein subclasses	mmol/l	0.0939	0.0948	0.0957	0.0462	0.0419	0.0444	0.0808	0.0851	0.0852	0.0667	0.0673	0.0648	0.1210	0.1118	0.1143
Cholesterol in very large VLDL	Lipoprotein subclasses	mmol/l	0.0602	0.0446	0.0482	0.0449	0.0251	0.0341	0.0472	0.0405	0.0431	0.0310	0.0270	0.0237	0.0739	0.0578	0.0688
Cholesterol in very small VLDL	Lipoprotein subclasses	mmol/l	0.1704	0.1600	0.1727	0.0443	0.0555	0.0511	0.1674	0.1551	0.1708	0.1393	0.1210	0.1392	0.1988	0.1835	0.1960
Cholesteryl esters in chylomicrons and extremely large VLDL	Lipoprotein subclasses	mmol/l	0.0340	0.0241	0.0216	0.0327	0.0154	0.0214	0.0242	0.0207	0.0169	0.0159	0.0138	0.0063	0.0449	0.0351	0.0280
Cholesteryl esters in IDL	Lipoprotein subclasses	mmol/l	0.6781	0.6083	0.6485	0.1668	0.1970	0.2183	0.6703	0.5878	0.6294	0.5564	0.4597	0.5104	0.8010	0.7362	0.7959
Cholesteryl esters in large HDL	Lipoprotein subclasses	mmol/l	0.2966	0.2890	0.2904	0.1612	0.1470	0.1681	0.2756	0.2652	0.2639	0.1866	0.1883	0.1654	0.3776	0.3740	0.3932
Cholesteryl esters in large LDL	Lipoprotein subclasses	mmol/l	0.9655	0.8381	0.9052	0.2550	0.2755	0.3361	0.9423	0.8366	0.9254	0.7956	0.6706	0.6668	1.1038	0.9800	1.1502
Cholesteryl esters in large VLDL	Lipoprotein subclasses	mmol/l	0.0530	0.0421	0.0465	0.0333	0.0230	0.0271	0.0453	0.0383	0.0428	0.0327	0.0256	0.0265	0.0637	0.0534	0.0669
Cholesteryl esters in medium HDL	Lipoprotein subclasses	mmol/l	0.4691	0.4357	0.4430	0.1017	0.1034	0.1279	0.4771	0.4266	0.4261	0.4008	0.3591	0.3391	0.5410	0.5091	0.5117
Cholesteryl esters in medium LDL	Lipoprotein subclasses	mmol/l	0.3669	0.3111	0.3293	0.1264	0.1051	0.1337	0.3504	0.2957	0.3358	0.2791	0.2394	0.2363	0.4382	0.3662	0.4352
Cholesteryl esters in medium VLDL	Lipoprotein subclasses	mmol/l	0.0911	0.0805	0.0848	0.0371	0.0451	0.0435	0.0903	0.0753	0.0789	0.0635	0.0487	0.0509	0.1137	0.1086	0.1182
Cholesteryl esters in small HDL	Lipoprotein subclasses	mmol/l	0.3663	0.3386	0.3414	0.0557	0.0544	0.0729	0.3613	0.3434	0.3512	0.3286	0.3041	0.2992	0.4017	0.3814	0.3885
Cholesteryl esters in small LDL	Lipoprotein subclasses	mmol/l	0.1519	0.1340	0.1374	0.0439	0.0384	0.0444	0.1453	0.1299	0.1450	0.1210	0.1105	0.1061	0.1756	0.1540	0.1729
Cholesteryl esters in small VLDL	Lipoprotein subclasses	mmol/l	0.0963	0.0908	0.0909	0.0355	0.0364	0.0327	0.0904	0.0845	0.0892	0.0743	0.0660	0.0657	0.1159	0.1052	0.1124
Cholesteryl esters in very large HDL	Lipoprotein subclasses	mmol/l	0.0706	0.0707	0.0709	0.0365	0.0331	0.0355	0.0620	0.0629	0.0639	0.0492	0.0491	0.0438	0.0900	0.0848	0.0873
Cholesteryl esters in very large VLDL	Lipoprotein subclasses	mmol/l	0.0341	0.0275	0.0280	0.0214	0.0139	0.0165	0.0284	0.0261	0.0258	0.0198	0.0171	0.0152	0.0417	0.0354	0.0391
Cholesteryl esters in very small VLDL	Lipoprotein subclasses	mmol/l	0.1159	0.1078	0.1173	0.0316	0.0393	0.0372	0.1138	0.1069	0.1150	0.0944	0.0804	0.0926	0.1373	0.1285	0.1368
Free cholesterol in chylomicrons and extremely large VLDL	Lipoprotein subclasses	mmol/l	0.0204	0.0104	0.0133	0.0282	0.0105	0.0198	0.0101	0.0068	0.0086	0.0022	0.0014	0.0010	0.0258	0.0165	0.0164
Free cholesterol in IDL	Lipoprotein subclasses	mmol/l	0.2240	0.2051	0.2163	0.0546	0.0674	0.0675	0.2269	0.1947	0.2105	0.1845	0.1601	0.1708	0.2508	0.2431	0.2684
Free cholesterol in large HDL	Lipoprotein subclasses	mmol/l	0.0830	0.0795	0.0838	0.0470	0.0433	0.0483	0.0741	0.0729	0.0726	0.0477	0.0478	0.0459	0.1082	0.1042	0.1116
Free cholesterol in large LDL	Lipoprotein subclasses	mmol/l	0.3347	0.2985	0.3151	0.0837	0.0991	0.1149	0.3284	0.2862	0.3058	0.2663	0.2318	0.2396	0.3878	0.3596	0.4021
Free cholesterol in large VLDL	Lipoprotein subclasses	mmol/l	0.0543	0.0391	0.0452	0.0416	0.0226	0.0331	0.0430	0.0338	0.0386	0.0259	0.0240	0.0225	0.0658	0.0523	0.0663
Free cholesterol in medium HDL	Lipoprotein subclasses	mmol/l	0.1028	0.0925	0.0981	0.0250	0.0260	0.0329	0.1035	0.0872	0.0925	0.0834	0.0745	0.0777	0.1149	0.1108	0.1151
Free cholesterol in medium LDL	Lipoprotein subclasses	mmol/l	0.1412	0.1252	0.1313	0.0383	0.0410	0.0506	0.1396	0.1218	0.1371	0.1153	0.1001	0.0972	0.1590	0.1491	0.1682
Free cholesterol in medium VLDL	Lipoprotein subclasses	mmol/l	0.0864	0.0712	0.0771	0.0369	0.0330	0.0349	0.0766	0.0650	0.0771	0.0653	0.0453	0.0455	0.1070	0.0881	0.1041
Free cholesterol in small HDL	Lipoprotein subclasses	mmol/l	0.1313	0.1212	0.1257	0.0188	0.0181	0.0245	0.1304	0.1222	0.1282	0.1163	0.1091	0.1062	0.1421	0.1318	0.1409
Free cholesterol in small LDL	Lipoprotein subclasses	mmol/l	0.0587	0.0537	0.0574	0.0147	0.0152	0.0189	0.0583	0.0525	0.0594	0.0492	0.0442	0.0436	0.0668	0.0610	0.0700
Free cholesterol in small VLDL	Lipoprotein subclasses	mmol/l	0.0632	0.0567	0.0588	0.0219	0.0220	0.0215	0.0599	0.0527	0.0583	0.0498	0.0410	0.0409	0.0739	0.0674	0.0753
Free cholesterol in very large HDL	Lipoprotein subclasses	mmol/l	0.0233	0.0241	0.0248	0.0099	0.0091	0.0095	0.0210	0.0223	0.0229	0.0170	0.0182	0.0186	0.0292	0.0277	0.0309
Free cholesterol in very large VLDL	Lipoprotein subclasses	mmol/l	0.0261	0.0171	0.0202	0.0239	0.0117	0.0183	0.0191	0.0141	0.0159	0.0106	0.0091	0.0088	0.0301	0.0243	0.0293
Free cholesterol in very small VLDL	Lipoprotein subclasses	mmol/l	0.0544	0.0522	0.0554	0.0137	0.0168	0.0152	0.0539	0.0496	0.0547	0.0446	0.0433	0.0461	0.0635	0.0586	0.0618
Phospholipids in chylomicrons and extremely large VLDL	Lipoprotein subclasses	mmol/l	0.0299	0.0139	0.0189	0.0458	0.0166	0.0326	0.0129	0.0069	0.0091	0.0002	0.0001	0.0002	0.0368	0.0243	0.0226
Phospholipids in IDL	Lipoprotein subclasses	mmol/l	0.2981	0.2762	0.2926	0.0679	0.0845	0.0854	0.2987	0.2685	0.2793	0.2418	0.2210	0.2333	0.3413	0.3132	0.3539
Phospholipids in large HDL	Lipoprotein subclasses	mmol/l	0.3840	0.3675	0.3855	0.1869	0.1702	0.1970	0.3600	0.3284	0.3456	0.2552	0.2495	0.2335	0.4856	0.4726	0.4968
Phospholipids in large LDL	Lipoprotein subclasses	mmol/l	0.3961	0.3569	0.3747	0.0932	0.1048	0.1193	0.3908	0.3502	0.3873	0.3277	0.2909	0.2922	0.4419	0.4154	0.4637
Phospholipids in large VLDL	Lipoprotein subclasses	mmol/l	0.0811	0.0575	0.0685	0.0668	0.0352	0.0549	0.0661	0.0499	0.0570	0.0357	0.0317	0.0315	0.0947	0.0809	0.0987
Phospholipids in medium HDL	Lipoprotein subclasses	mmol/l	0.5252	0.4787	0.5042	0.0960	0.0946	0.1288	0.5257	0.4712	0.4906	0.4577	0.4054	0.4197	0.5841	0.5583	0.5797
Phospholipids in medium LDL	Lipoprotein subclasses	mmol/l	0.1922	0.1709	0.1781	0.0534	0.0510	0.0612	0.1882	0.1643	0.1853	0.1566	0.1408	0.1367	0.2165	0.2012	0.2247
Phospholipids in medium VLDL	Lipoprotein subclasses	mmol/l	0.1449	0.1187	0.1302	0.0646	0.0520	0.0588	0.1282	0.1100	0.1274	0.1074	0.0840	0.0851	0.1745	0.1419	0.1738
Phospholipids in small HDL	Lipoprotein subclasses	mmol/l	0.7047	0.6424	0.6625	0.1073	0.0905	0.1336	0.6912	0.6430	0.6745	0.6247	0.5694	0.5725	0.7680	0.6968	0.7447
Phospholipids in small LDL	Lipoprotein subclasses	mmol/l	0.0999	0.0934	0.0982	0.0227	0.0223	0.0247	0.0981	0.0946	0.0994	0.0853	0.0768	0.0792	0.1107	0.1029	0.1159

Phospholipids in small VLDL	Lipoprotein subclasses	mmol/l	0.1055	0.0947	0.0987	0.0366	0.0333	0.0331	0.0979	0.0885	0.0961	0.0834	0.0728	0.0737	0.1238	0.1113	0.1280
Phospholipids in very large HDL	Lipoprotein subclasses	mmol/l	0.0879	0.0897	0.0907	0.0605	0.0530	0.0594	0.0730	0.0792	0.0691	0.0455	0.0564	0.0500	0.1277	0.1121	0.1181
Phospholipids in very large VLDL	Lipoprotein subclasses	mmol/l	0.0443	0.0276	0.0345	0.0438	0.0206	0.0357	0.0313	0.0226	0.0248	0.0157	0.0135	0.0123	0.0501	0.0434	0.0505
Phospholipids in very small VLDL	Lipoprotein subclasses	mmol/l	0.0978	0.0968	0.1018	0.0251	0.0309	0.0290	0.0942	0.0910	0.0965	0.0780	0.0789	0.0843	0.1120	0.1088	0.1164
Total lipids in chylomicrons and extremely large VLDL	Lipoprotein subclasses	mmol/l	0.2279	0.1044	0.1548	0.3233	0.1180	0.2775	0.0919	0.0448	0.0648	0.0217	0.0165	0.0094	0.2820	0.1576	0.1859
Total lipids in IDL	Lipoprotein subclasses	mmol/l	1.3069	1.1968	1.2709	0.2984	0.3602	0.3768	1.3113	1.1585	1.2241	1.0662	0.9334	1.0063	1.5285	1.4039	1.5325
Total lipids in large HDL	Lipoprotein subclasses	mmol/l	0.7933	0.7623	0.7908	0.4003	0.3645	0.4218	0.7391	0.6960	0.7119	0.5045	0.5029	0.4665	1.0155	0.9735	1.0362
Total lipids in large LDL	Lipoprotein subclasses	mmol/l	1.8054	1.6029	1.7101	0.4443	0.4914	0.5752	1.7686	1.6083	1.7534	1.4967	1.2921	1.3018	2.0427	1.8638	2.1048
Total lipids in large VLDL	Lipoprotein subclasses	mmol/l	0.4203	0.2946	0.3495	0.3167	0.1656	0.2769	0.3363	0.2560	0.2802	0.2043	0.1826	0.1709	0.4817	0.4229	0.4928
Total lipids in medium HDL	Lipoprotein subclasses	mmol/l	1.1472	1.0491	1.0943	0.2216	0.2220	0.2939	1.1573	1.0290	1.0575	0.9908	0.8780	0.8959	1.2752	1.2436	1.2463
Total lipids in medium LDL	Lipoprotein subclasses	mmol/l	0.7389	0.6441	0.6779	0.2220	0.2004	0.2481	0.7140	0.6132	0.7034	0.5938	0.5177	0.5155	0.8395	0.7629	0.8667
Total lipids in medium VLDL	Lipoprotein subclasses	mmol/l	0.6746	0.5414	0.5998	0.3126	0.2188	0.2828	0.5988	0.4956	0.5692	0.4732	0.3957	0.3854	0.7825	0.6110	0.7868
Total lipids in small HDL	Lipoprotein subclasses	mmol/l	1.2558	1.1499	1.1816	0.1901	0.1629	0.2336	1.2204	1.1450	1.1981	1.1272	1.0437	1.0252	1.3655	1.2548	1.3175
Total lipids in small LDL	Lipoprotein subclasses	mmol/l	0.3282	0.2966	0.3100	0.0840	0.0771	0.0898	0.3203	0.2877	0.3191	0.2750	0.2531	0.2421	0.3631	0.3322	0.3751
Total lipids in small VLDL	Lipoprotein subclasses	mmol/l	0.4464	0.3993	0.4205	0.1659	0.1345	0.1401	0.4110	0.3693	0.4105	0.3529	0.3114	0.3141	0.5153	0.4559	0.5160
Total lipids in very large HDL	Lipoprotein subclasses	mmol/l	0.1896	0.1916	0.1947	0.1068	0.0956	0.1047	0.1581	0.1673	0.1612	0.1185	0.1319	0.1161	0.2600	0.2251	0.2387
Total lipids in very large VLDL	Lipoprotein subclasses	mmol/l	0.2538	0.1619	0.2028	0.2328	0.1105	0.2076	0.1796	0.1348	0.1469	0.0978	0.0884	0.0746	0.2924	0.2522	0.2853
Total lipids in very small VLDL	Lipoprotein subclasses	mmol/l	0.3417	0.3293	0.3511	0.0826	0.0998	0.0937	0.3307	0.3099	0.3409	0.2815	0.2704	0.2944	0.3935	0.3655	0.3907
Triglycerides in chylomicrons and extremely large VLDL	Lipoprotein subclasses	mmol/l	0.1435	0.0560	0.1009	0.2181	0.0780	0.2064	0.0448	0.0120	0.0226	0.0062	0.0001	0.0000	0.1868	0.0892	0.1117
Triglycerides in IDL	Lipoprotein subclasses	mmol/l	0.1067	0.1072	0.1135	0.0243	0.0267	0.0317	0.1038	0.1029	0.1060	0.0884	0.0919	0.0931	0.1191	0.1167	0.1256
Triglycerides in large HDL	Lipoprotein subclasses	mmol/l	0.0297	0.0264	0.0311	0.0127	0.0114	0.0156	0.0262	0.0247	0.0267	0.0207	0.0192	0.0203	0.0366	0.0319	0.0373
Triglycerides in large LDL	Lipoprotein subclasses	mmol/l	0.1091	0.1094	0.1151	0.0247	0.0266	0.0315	0.1079	0.1063	0.1083	0.0913	0.0944	0.0944	0.1221	0.1197	0.1282
Triglycerides in large VLDL	Lipoprotein subclasses	mmol/l	0.2319	0.1560	0.1893	0.1768	0.0886	0.1655	0.1807	0.1316	0.1456	0.1134	0.0946	0.0818	0.2744	0.2092	0.2594
Triglycerides in medium HDL	Lipoprotein subclasses	mmol/l	0.0500	0.0421	0.0490	0.0204	0.0163	0.0209	0.0460	0.0416	0.0447	0.0349	0.0319	0.0350	0.0638	0.0493	0.0581
Triglycerides in medium LDL	Lipoprotein subclasses	mmol/l	0.0386	0.0369	0.0391	0.0113	0.0093	0.0117	0.0366	0.0351	0.0362	0.0315	0.0314	0.0311	0.0438	0.0406	0.0456
Triglycerides in medium VLDL	Lipoprotein subclasses	mmol/l	0.3522	0.2711	0.3076	0.1998	0.1142	0.1782	0.2951	0.2550	0.2755	0.2134	0.1866	0.1892	0.4098	0.3517	0.4131
Triglycerides in small HDL	Lipoprotein subclasses	mmol/l	0.0535	0.0478	0.0519	0.0219	0.0156	0.0197	0.0484	0.0482	0.0485	0.0389	0.0378	0.0383	0.0646	0.0587	0.0621
Triglycerides in small LDL	Lipoprotein subclasses	mmol/l	0.0176	0.0155	0.0170	0.0072	0.0043	0.0067	0.0157	0.0148	0.0155	0.0127	0.0125	0.0128	0.0192	0.0173	0.0192
Triglycerides in small VLDL	Lipoprotein subclasses	mmol/l	0.1814	0.1571	0.1721	0.0826	0.0568	0.0704	0.1601	0.1548	0.1594	0.1248	0.1158	0.1245	0.2103	0.1888	0.2127
Triglycerides in very large HDL	Lipoprotein subclasses	mmol/l	0.0078	0.0071	0.0082	0.0027	0.0026	0.0034	0.0073	0.0071	0.0074	0.0057	0.0053	0.0059	0.0094	0.0084	0.0098
Triglycerides in very large VLDL	Lipoprotein subclasses	mmol/l	0.1493	0.0897	0.1201	0.1451	0.0674	0.1400	0.0967	0.0704	0.0784	0.0520	0.0452	0.0333	0.1807	0.1324	0.1620
Triglycerides in very small VLDL	Lipoprotein subclasses	mmol/l	0.0735	0.0724	0.0766	0.0221	0.0214	0.0249	0.0715	0.0703	0.0697	0.0573	0.0579	0.0599	0.0870	0.0822	0.0893
Phospholipids in HDL	Phospholipids	mmol/l	1.7019	1.5782	1.6430	0.3274	0.3310	0.4173	1.6988	1.5190	1.5622	1.4521	1.3498	1.3710	1.8652	1.8211	1.9177
Phospholipids in LDL	Phospholipids	mmol/l	0.6883	0.6213	0.6511	0.1662	0.1766	0.2024	0.6760	0.6213	0.6796	0.5701	0.5153	0.5090	0.7765	0.7137	0.7958
Phospholipids in VLDL	Phospholipids	mmol/l	0.5035	0.4093	0.4528	0.2596	0.1662	0.2076	0.4355	0.3801	0.4196	0.3433	0.2873	0.3004	0.5631	0.4822	0.5744
Total phospholipids in lipoprotein particles	Phospholipids	mmol/l	3.1918	2.8849	3.0394	0.4951	0.5493	0.6469	3.1884	2.8139	3.0191	2.7763	2.5370	2.5661	3.5169	3.2244	3.4705
Total lipids in HDL	Total lipids	mmol/l	3.3859	3.1530	3.2613	0.6753	0.6911	0.8493	3.3679	3.0145	3.0782	2.8308	2.7001	2.6751	3.7315	3.6519	3.8190
Total lipids in LDL	Total lipids	mmol/l	2.8724	2.5436	2.6980	0.7345	0.7631	0.9035	2.8290	2.5464	2.8015	2.3998	2.0416	2.0660	3.2551	2.9678	3.3347
Total lipids in lipoprotein particles	Total lipids	mmol/l	9.9299	8.7243	9.3088	2.0206	1.8926	2.3186	9.5083	8.5405	9.4457	8.4520	7.5477	7.4615	11.1220	9.7301	10.8988
Total lipids in VLDL	Total lipids	mmol/l	2.3646	1.8309	2.0785	1.3507	0.7519	1.1399	2.0270	1.7182	1.8556	1.4830	1.2702	1.2852	2.5975	2.2345	2.6607

Supplementary table 2

Name	Group	Cohort	Mean value Box-Cox transformed adjusted for covariates	ANCOVA F-value	ANCOVA p-value	Sidak pairwise comparision p-value, Compared to CTRL	Sidak pairwise comparision p-value, Compared to LS vs CRC	Levene's test	GLiM wald Chi-square	GLiM Wald Chi-square sig.	GLiM Pairwise comparision, with a sequential Sidak correction, agains CTRL	GLiM Pairwise comparision, with a sequential Sidak correction, LS vs CRC
Apolipoprotein A1	Apolipoproteins	CTRL	0.41					Unequal variance p=0.020	9.478	**0.009**		
		LS	0.472								**0.042**	**0.042**
		CRC	0.391								0.933	
Apolipoprotein B	Apolipoproteins	CTRL	-0.19	2.052	0.131							
		LS	-0.109									
		CRC	-0.18									
ApoB/ApoA1	Apolipoproteins	CTRL	-0.634	0.288	0.75							
		LS	-0.608									
		CRC	-0.599									
Total cholesterol	Cholesterols	CTRL	3.069	3.645	**0.028**							
		LS	3.382			0.063	**0.047**					
		CRC	3.027			0.986						
VLDL cholesterol	Cholesterols	CTRL	-0.494	2.322	0.1							
		LS	-0.374									
		CRC	-0.465									
Remnant cholesterol	Cholesterols	CTRL	0.387	2.659	0.072							
		LS	0.509									
		CRC	0.386									
LDL cholesterol	Cholesterols	CTRL	0.724					Unequal variance p=0.005	9.611	**0.008**		
		LS	0.914								0.055	**0.008**
		CRC	0.737								0.852	
HDL cholesterol	Cholesterols	CTRL	0.359	2.435	0.09							
		LS	0.418									
		CRC	0.33									
VLDL diameter	Lipoprotein particle size	CTRL	0.5					Unequal variance p=0.025	6.314	**0.043**		
		LS	0.5								0.78	0.76
		CRC	0.5								1	
LDL diameter	Lipoprotein particle size	CTRL	285.413					Unequal variance p=0.013	1.307	0.52		
		LS	284.953									
		CRC	285.109									
HDL diameter	Lipoprotein particle size	CTRL	0.495	1.165	0.314							
		LS	0.495									
		CRC	0.495									
Total VLDL	Lipoprotein particles	CTRL	-8.171	1.517	0.222							
		LS	-8.102									
		CRC	-8.171									
Extremely large VLDL	Lipoprotein particles	CTRL	-7.746	2.723	0.068							
		LS	-7.602									
		CRC	-7.691									
Extra large VLDL	Lipoprotein particles	CTRL	-3.235	2.591	0.077							
		LS	-3.229									
		CRC	-3.233									
Large VLDL	Lipoprotein particles	CTRL	-6.877	2.54	0.081							
		LS	-6.809									
		CRC	-6.858									
Medium VLDL	Lipoprotein particles	CTRL	-3.559	2.904	0.057							
		LS	-3.549									
		CRC	-3.557									
Small VLDL	Lipoprotein particles	CTRL	-4.638	1.332	0.266							
		LS	-4.625									
		CRC	-4.635									
very small VLDL	Lipoprotein particles	CTRL	-27.604	0.769	0.465							
		LS	-27.571									
		CRC	-27.858									
Total IDL	Lipoprotein particles	CTRL	-2.49	1.704	0.184							
		LS	-2.487									
		CRC	-2.491									
Total LDL	Lipoprotein particles	CTRL	-3.16	2.076	0.128							
		LS	-3.145									
		CRC	-3.158									
Large LDL	Lipoprotein particles	CTRL	-4.532	1.675	0.189							
		LS	-4.504									
		CRC	-4.528									
Medium LDL	Lipoprotein particles	CTRL	-2.104					Unequal variance p=0.01	6.722	**0.035**		
		LS	-2.101								0.077	0.055
		CRC	-2.103								0.997	
Small LDL	Lipoprotein particles	CTRL	-3.062	2.285	0.104			Unequal variance p=0.032	6.734	**0.034**		
		LS	-3.056								0.119	**0.039**
		CRC	-3.061								0.945	
Total HDL	Lipoprotein particles	CTRL	-1.405	4.915	0.008			Unequal variance p=0.004	12.45	**0.002**		
		LS	-1.4								**0.014**	**0.003**
		CRC	-1.406								0.916	
Extra large HDL	Lipoprotein particles	CTRL	-10.691	1.147	0.319							
		LS	-10.749									
		CRC	-10.841									
Large HDL	Lipoprotein particles	CTRL	-2.827	0.913	0.403							
		LS	-2.825									
		CRC	-2.84									
Medium HDL	Lipoprotein particles	CTRL	-2.474					Unequal variance p=0.030	8.955	**0.011**		
		LS	-2.461								0.053	**0.13**
		CRC	-2.478								0.929	
Small HDL	Lipoprotein particles	CTRL	-0.783					Unequal variance p=0.011	13.305	**0.001**		
		LS	-0.783								**0.008**	**0.002**
		CRC	-0.783								0.962	
Total Triglycerides	Triglycerides	CTRL	119	2.335	0.099							
		LS	0.23									
		CRC	0.107									
VLDL triglycerides	Triglycerides	CTRL	-0.357	3.284	**0.039**							
		LS	-0.141			**0.044**	0.145					
		CRC	-0.328			0.983						
LDL triglycerides	Triglycerides	CTRL	-2.856	0.781	0.459							
		LS	-2.883									
		CRC	-2.963									
HDL triglycerides	Triglycerides	CTRL	-2.098	1.583	0.207							
		LS	-2.07									
		CRC	-2.171									
Alanine	Aminoacids	CTRL	-0.897	3.867	**0.022**							
		LS	-0.945			0.52	0.403					
		CRC	-1.004			**0.018**						
Glutamine	Aminoacids	CTRL	-0.281	11.804	**0.001**							
		LS	-0.222			**0**	0.851					
		CRC	-0.232			**0**						
Glycine	Aminoacids	CTRL	-1.718	6.358	**0.002**							
		LS	-1.899			0.061	0.68					
		CRC	-1.983			**0.002**						
Histidine	Aminoacids	CTRL	-1.122	41.712	**0.001**							
		LS	-1.137			**0**	**0**					
		CRC	-1.152			**0**						
Isoleucine	Aminoacids	CTRL	-4.978	5.453	**0.005**							
		LS	-5.391			**0.006**	0.816					
		CRC	-5.278			0.07						
Leucine	Aminoacids	CTRL	-1.82	13.987	**0.001**							
		LS	-1.908			**0.032**	**0.041**					
		CRC	-2			**0**						
Phenylalanine	Aminoacids	CTRL	-8.349					Unequal variance p=0.006	87.939	**0**		
		LS	-9.908								**0**	**0.01**
		CRC	-10.605								**0**	
Total BCAA	Aminoacids	CTRL	-0.826	12.923	0.001							
		LS	-0.929			**0.023**	0.95					

		CRC	-1.018			**0**						
Tyrosine	Aminoacids	CTRL	-4.136					Unequal variance p=0.032	63.475	**0**		
		LS	-4.502								**0**	**0.002**
		CRC	-4.844								**0**	
Valine	Aminoacids	CTRL	-1.345	13.364	0.001							
		LS	-1.424			**0.04**	**0.047**					
		CRC	-1.508			**0**						
Total fatty acids	Fatty Acids rations	CTRL	1.676					Unequal variance p=0.011	15.616	**0**		
		LS	1.729								**0.001**	**0.005**
		CRC	1.692								0.904	
DHA %	Fatty Acids rations	CTRL	1.064	1.642	0.196							
		LS	0.975									
		CRC	1.014									
Linoleic acid %	Fatty Acids rations	CTRL	435.74					Unequal variance p=0.001	21.486	**0**		
		LS	501.034								**0.001**	0.996
		CRC	508.049								**0**	
Omega-3 %	Fatty Acids rations	CTRL	1.978	0.351	0.704							
		LS	1.988									
		CRC	1.936									
Omega-6 %	Fatty Acids rations	CTRL	657.06	1.415	0.245							
		LS	673.733									
		CRC	688.99									
PUFA %	Fatty Acids rations	CTRL	852.851	0.912	0.403							
		LS	871.837									
		CRC	882.196									
MUFA %	Fatty Acids rations	CTRL	1.215	1.299	0.275							
		LS	1.217									
		CRC	1.217									
SFA %	Fatty Acids rations	CTRL	0.5					Unequal variance p=0.016	28.798	**0**		
		LS	0.5								**0.001**	0.423
		CRC	0.5								**0**	
Glucose	Glycolysis related metabolite	CTRL	0.87	6.893	0.001							
		LS	0.878			0.517	**0.072**					
		CRC	0.894			**0**						
Pyruvate	Glycolysis related metabolite	CTRL	-2.866					Unequal variance p=0.001	55.466	**0**		
		LS	-2.63								**0.002**	**0.001**
		CRC	-2.425								**0**	
Citrate	Glycolysis related metabolite	CTRL	-4.933	1.298	0.275							
		LS	-4.986									
		CRC	-4.853									
Acetate	Ketone bodies	CTRL	-12.039					Unequal variance p=0.044	27.648	**0**		
		LS	-15.156								**0**	0.706
		CRC	-14.564								**0**	
Acetoacetate	Ketone bodies	CTRL	-3.509	40.759	0.001							
		LS	-2.674			**0**	0.547					
		CRC	-2.521			**0**						
Acetone	Ketone bodies	CTRL	-14.447	4.107	0.018							
		LS	-13.915			0.821	0.169					
		CRC	-12.52			**0.015**						
3-hydroxybutyrate	Ketone bodies	CTRL	-2.713					Unequal variance p=0.020	35.266	**0**		
		LS	-2.191								**0.003**	0.079
		CRC	-1.851								**0**	
Albumin	Miscellaneous	CTRL	808.502	1.503	0.224							
		LS	862.493									
		CRC	816.22									
Creatinine	Miscellaneous	CTRL	2.409	1.71	0.183							
		LS	2.424									
		CRC	2.413									
Glycoprotein acetyls (G	Miscellaneous	CTRL	-0.17	13.28	0.001							
		LS	-0.111			0.079	**0.023**					
		CRC	-0.034			**0**						
Total cholines	Phospholipids	CTRL	1.305					Unequal variance p=0.004	16.169	**0**		
		LS	1.475								**0.001**	**0.002**
		CRC	1.327								0.993	
Phosphatidylcholines	Phospholipids	CTRL	0.93					Unequal variance p=0.004	15.85	**0**		
		LS	1.047								**0.004**	**0**
		CRC	0.917								0.877	
Phosphoglycerides	Phospholipids	CTRL	1.052					Unequal variance p=0.004	17.081	**0**		
		LS	1.209								**0.001**	**0.001**
		CRC	1.068								0.999	
Sphingomyelins	Phospholipids	CTRL	-0.544	1.309	0.272							
		LS	-0.519									
		CRC	-0.541									
TG/PG ration	Phospholipids	CTRL	-0.81	0.206	0.814							
		LS	-0.799									
		CRC	-0.846									

Supplementary table 3

Name	Group	Variant	Mean value non transformed	unit	Sd value non transformed	Mean value Box-Cox transformed adjusted for	ANCOVA F-value	ANCOVA p-value	Sidak pairwise comparision p-value, Compared to MLH1	Sidak pairwise comparision p-value, Compared to MSH2	Levene's test	GLiM wald Chi-square		GLiM Pairwise comparision, with a sequential Sidak correction, against MLH1 cohort	GLiM Pairwise comparision, with a sequential Sidak correction, against MSH2
Apolipoprotein A1	Apolipoproteins	MLH1	1.661	g/l	0.250	0.500	1.943	0.132							
		MSH2	1.522	g/l	0.235	0.434									
		MSH6	1.565	g/l	0.189	0.452									
		PMS2	1.181	g/l	NA	0.215									
Apolipoprotein B	Apolipoproteins	MLH1	0.955	g/l	0.223	-0.029					Unequal variance p= 0.031	6.423	**0.04**		
		MSH2	0.868	g/l	0.317	-0.166								0.237	
		MSH6	0.822	g/l	0.149	-0.257								0.085	0.998
		PMS2	0.796	g/l	NA	-0.266									
ApoB/ApoA1	Apolipoproteins	MLH1	0.587	ratio	0.167	-0.548					Unequal variance p= 0.028	1.731	0.421		
		MSH2	0.601	ratio	0.274	-0.648									
		MSH6	0.533	ratio	0.119	-0.742									
		PMS2	0.675	ratio	NA	-0.495									
Total cholesterol	Cholesterols	MLH1	5.440	mmol/l	0.984	3.645	6.361	**0.003**							
		MSH2	4.808	mmol/l	1.075	3.212			0.188						
		MSH6	4.724	mmol/l	0.718	2.938			**0.004**	0.706					
		PMS2	3.933	mmol/l	NA	2.436									
VLDL cholesterol	Cholesterols	MLH1	0.761	mmol/l	0.285	-0.272	4.063	**0.022**							
		MSH2	0.725	mmol/l	0.448	-0.436			0.465						
		MSH6	0.622	mmol/l	0.188	-0.578			**0.024**	0.72					
		PMS2	0.656	mmol/l	NA	-0.496									
Remnant cholesterol	Cholesterols	MLH1	1.723	mmol/l	0.430	0.629	6.429	**0.003**							
		MSH2	1.527	mmol/l	0.572	0.441			0.252						
		MSH6	1.416	mmol/l	0.294	0.287			**0.003**	**0.581**					
		PMS2	1.243	mmol/l	NA	0.186									
LDL cholesterol	Cholesterols	MLH1	2.125	mmol/l	0.527	1.064	5.121	**0.009**							
		MSH2	1.834	mmol/l	0.684	0.815			0.266						
		MSH6	1.819	mmol/l	0.350	0.660			**0.012**	**0.775**					
		PMS2	1.721	mmol/l	NA	0.578									
HDL cholesterol	Cholesterols	MLH1	1.592	mmol/l	0.355	0.446	1.572	0.205							
		MSH2	1.447	mmol/l	0.423	0.364									
		MSH6	1.489	mmol/l	0.255	0.416									
		PMS2	0.969	mmol/l	NA	0.055									
Total Triglycerides	Triglycerides	MLH1	1.567	mmol/l	0.836	0.295	1.293	0.284							
		MSH2	1.695	mmol/l	1.474	0.191									
		MSH6	1.314	mmol/l	0.504	0.062									
		PMS2	1.638	mmol/l	NA	0.307									
VLDL diameter	Lipoprotein particle si	MLH1	38.882	nm	1.586	0.500	0.711	0.549							
		MSH2	38.785	nm	2.560	0.500									
		MSH6	38.883	nm	1.078	0.500									
		PMS2	40.945	nm	NA	0.500									
LDL diameter	Lipoprotein particle si	MLH1	23.900	nm	0.089	285.038	2.078	0.112							
		MSH2	23.879	nm	0.122	285.124									
		MSH6	23.888	nm	0.051	285.015									
		PMS2	23.679	nm	NA	280.308									
HDL diameter	Lipoprotein particle si	MLH1	9.708	nm	0.241	0.495	0.518	0.671							
		MSH2	9.726	nm	0.335	0.495									
		MSH6	9.651	nm	0.149	0.495									
		PMS2	9.364	nm	NA	0.494									
Total VLDL	Lipoprotein particles	MLH1	0.00016	mmol/l	5.424e-05	-8.026	2.806	0.047							
		MSH2	0.00015	mmol/l	8.410e-05	-8.146			0.77						
		MSH6	0.00013	mmol/l	3.474e-05	-8.268			**0.044**	0.883					
		PMS2	0.00013	mmol/l	NA	-8.230									
Extremely large VLDL	Lipoprotein particles	MLH1	1.538e-06	mmol/l	1.901e-06	-7.574	0.466	0.707							
		MSH2	2.400e-06	mmol/l	4.136e-06	-7.624									
		MSH6	1.148e-06	mmol/l	1.174e-06	-7.690									
		PMS2	2.819e-06	mmol/l	NA	-7.310									
Extra large VLDL	Lipoprotein particles	MLH1	4.286e-06	mmol/l	3.514e-06	-3.227	0.880	0.456							
		MSH2	5.052e-06	mmol/l	6.320e-06	-3.233									
		MSH6	3.363e-06	mmol/l	2.022e-06	-3.230									
		PMS2	5.495e-06	mmol/l	NA	-3.215									
Large VLDL	Lipoprotein particles	MLH1	1.288e-05	mmol/l	8.901e-06	-6.782	1.134	0.342							
		MSH2	1.425e-05	mmol/l	1.528e-05	-6.874									
		MSH6	1.030e-05	mmol/l	4.940e-06	-6.827									
		PMS2	1.468e-05	mmol/l	NA	-6.683									
Medium VLDL	Lipoprotein particles	MLH1	4.056e-05	mmol/l	1.630e-05	-3.542	2.659	0.056							
		MSH2	3.850e-05	mmol/l	2.532e-05	-3.558									
		MSH6	3.295e-05	mmol/l	1.009e-05	-3.556									
		PMS2	3.466e-05	mmol/l	NA	-3.551									
Small VLDL	Lipoprotein particles	MLH1	4.458e-05	mmol/l	1.603e-05	-4.615	1.786	0.159							
		MSH2	4.218e-05	mmol/l	2.401e-05	-4.642									
		MSH6	3.914e-05	mmol/l	1.057e-05	-4.635									
		PMS2	3.717e-05	mmol/l	NA	-4.639									
very small VLDL	Lipoprotein particles	MLH1	5.603e-05	mmol/l	1.245e-05	-27.173	5.369	0.002							
		MSH2	5.017e-05	mmol/l	1.404e-05	-27.831			0.688						
		MSH6	4.645e-05	mmol/l	7.903e-06	-28.351			**0.002**	0.472					
		PMS2	3.915e-05	mmol/l	NA	-29.308									
Total IDL	Lipoprotein particles	MLH1	0.00034	mmol/l	6.929e-05	-2.484	4.926	0.004							
		MSH2	0.00029	mmol/l	8.089e-05	-2.491			0.173						
		MSH6	0.00028	mmol/l	5.301e-05	-2.492			**0.010**	0.987					
		PMS2	0.00024	mmol/l	NA	-2.498									
Total LDL	Lipoprotein particles	MLH1	0.00137	mmol/l	0.00033	-3.133					Unequal variance p= 0.023	5.557	0.062		
		MSH2	0.00125	mmol/l	0.00047	-3.159									
		MSH6	0.00119	mmol/l	0.00022	-3.160									
		PMS2	0.00118	mmol/l	NA	-3.160									
Large LDL	Lipoprotein particles	MLH1	0.00083	mmol/l	0.00019	-4.475					Unequal variance p= 0.020	5.696	0.058		
		MSH2	0.00075	mmol/l	0.00028	-4.524									
		MSH6	0.00072	mmol/l	0.00012	-4.556									
		PMS2	0.00063	mmol/l	NA	-4.591									
Medium LDL	Lipoprotein particles	MLH1	0.00034	mmol/l	0.00010	-2.099					Unequal variance p= 0.030	4.373	0.112		
		MSH2	0.00031	mmol/l	0.00014	-2.103									
		MSH6	0.00030	mmol/l	6.415e-05	-2.102									
		PMS2	0.00036	mmol/l	NA	-2.098									
Small LDL	Lipoprotein particles	MLH1	0.00020	mmol/l	4.651e-05	-3.051					Unequal variance p= 0.038	5.04	0.08		
		MSH2	0.00018	mmol/l	6.670e-05	-3.059									
		MSH6	0.00017	mmol/l	3.042e-05	-3.066									
		PMS2	0.00018	mmol/l	NA	-3.062									
Total HDL	Lipoprotein particles	MLH1	0.01770	mmol/l	0.00248	-1.398	2.482	0.069							
		MSH2	0.01580	mmol/l	0.00168	-1.404									
		MSH6	0.01683	mmol/l	0.00209	-1.401									
		PMS2	0.01307	mmol/l	NA	-1.415									
Extra large HDL	Lipoprotein particles	MLH1	0.00028	mmol/l	0.00013	-10.705	1.029	0.386							
		MSH2	0.00029	mmol/l	0.00015	-10.662									
		MSH6	0.00023	mmol/l	7.484e-05	-10.869									
		PMS2	0.00014	mmol/l	NA	-11.547									
Large HDL	Lipoprotein particles	MLH1	0.00188	mmol/l	0.00096	-2.821	0.943	0.425							
		MSH2	0.00179	mmol/l	0.00117	-2.830									
		MSH6	0.00159	mmol/l	0.00058	-2.824									
		PMS2	0.00052	mmol/l	NA	-2.932									
Medium HDL	Lipoprotein particles	MLH1	0.00456	mmol/l	0.00093	-2.456	1.668	0.183							
		MSH2	0.00401	mmol/l	0.00091	-2.471									
		MSH6	0.00427	mmol/l	0.00074	-2.463									
		PMS2	0.00279	mmol/l	NA	-2.514									
Small HDL	Lipoprotein particles	MLH1	0.01098	mmol/l	0.00171	-0.783					Unequal variance p= 0.027	5.924	0.052		
		MSH2	0.00971	mmol/l	0.00130	-0.783									
		MSH6	0.01073	mmol/l	0.00127	-0.783									
		PMS2	0.00962	mmol/l	NA	-0.784									
Total Triglycerides	Triglycerides	MLH1	1.567	mmol/l	0.836	0.295	1.293	0.284							
		MSH2	1.695	mmol/l	1.474	0.191									
		MSH6	1.314	mmol/l	0.504	0.062									
		PMS2	1.638	mmol/l	NA	0.307									
VLDL triglycerides	Triglycerides	MLH1	1.140	mmol/l	0.757	-0.056	0.977	0.409							
		MSH2	1.290	mmol/l	1.329	-0.180									
		MSH6	0.942	mmol/l	0.442	-0.347									
		PMS2	1.307	mmol/l	NA	0.43									
LDL triglycerides	Triglycerides	MLH1	0.170	mmol/l	0.040	-2.775					Unequal variance p= 0.033	3.588	0.166		
		MSH2	0.161	mmol/l	0.061	-3.000									
		MSH6	0.152	mmol/l	0.024	-3.211									
		PMS2	0.138	mmol/l	NA	-3.286									
HDL triglycerides	Triglycerides	MLH1	0.147	mmol/l	0.047	-2.003					Unequal variance p= 0.011	2.609	0.271		
		MSH2	0.140	mmol/l	0.074	-2.080									
		MSH6	0.123	mmol/l	0.033	-2.237									
		PMS2	0.112	mmol/l	NA	-2.281									
Alanine	Aminoacids	MLH1	0.442	mmol/l	0.087	-0.939	0.429	0.733							
		MSH2	0.429	mmol/l	0.093	-0.934									
		MSH6	0.422	mmol/l	0.069	-1.1010									
		PMS2	0.399	mmol/l	NA	-1.029									
Glutamine	Aminoacids	MLH1	0.759	mmol/l	0.090	-0.223	0.272	0.845							
		MSH2	0.780	mmol/l	0.132	-0.246									
		MSH6	0.763	mmol/l	0.103	-0.225									
		PMS2	0.731	mmol/l	NA	-0.276									
Glycine	Aminoacids	MLH1	0.292	mmol/l	0.091	-1.853	0.906	0.443							
		MSH2	0.317	mmol/l	0.101	-1.813									

		MSH6	0.262	mmol/l	0.076	-2.095							
		PMS2	0.241	mmol/l	NA	-1.901							
Histidine	Aminoacids	MLH1	0.080	mmol/l	0.009	-1.134	0.795	0.501					
		MSH2	0.079	mmol/l	0.011	-1.142							
		MSH6	0.079	mmol/l	0.012	-1.136							
		PMS2	0.079	mmol/l	NA	-1.145							
Isoleucine	Aminoacids	MLH1	0.059	mmol/l	0.017	-5.270	0.644	0.590					
		MSH2	0.054	mmol/l	0.021	-5.669							
		MSH6	0.057	mmol/l	0.016	-5.490							
		PMS2	0.053	mmol/l	NA	-5.857							
Leucine	Aminoacids	MLH1	0.128	mmol/l	0.030	-1.876	1.088	0.361					
		MSH2	0.115	mmol/l	0.036	-1.989							
		MSH6	0.130	mmol/l	0.030	-1.870							
		PMS2	0.116	mmol/l	NA	-2.002							
Phenylalanine	Aminoacids	MLH1	0.072	mmol/l	0.012	-9.724	1.937	0.133					
		MSH2	0.069	mmol/l	0.014	-9.961							
		MSH6	0.069	mmol/l	0.008	-10.118							
		PMS2	0.052	mmol/l	NA	-12.714							
Total BCAA	Aminoacids	MLH1	0.430	mmol/l	0.088	-0.905	0.485	0.694					
		MSH2	0.409	mmol/l	0.110	-0.970							
		MSH6	0.436	mmol/l	0.092	-0.918							
		PMS2	0.379	mmol/l	NA	-1.109							
Tyrosine	Aminoacids	MLH1	0.069	mmol/l	0.015	-4.402	2.822	0.067					
		MSH2	0.064	mmol/l	0.010	-4.573							
		MSH6	0.063	mmol/l	0.011	-4.762							
		PMS2	0.046	mmol/l	NA	-5.483							
Valine	Aminoacids	MLH1	0.243	mmol/l	0.045	-1.411	0.580	0.631					
		MSH2	0.234	mmol/l	0.056	-1.443							
		MSH6	0.249	mmol/l	0.049	-1.415							
		PMS2	0.210	mmol/l	NA	-1.620							
Total fatty acids	Fatty Acids rations	MLH1	14.573	mmol/l	3.023	1.750					Unequal variance p= 0.041	4.695	0.096
		MSH2	13.941	mmol/l	4.551	1.719							
		MSH6	13.100	mmol/l	2.072	1.685							
		PMS2	12.150	mmol/l	NA	1.663							
DHA %	Fatty Acids rations	MLH1	2.358	%	0.506	0.972	0.328	0.805					
		MSH2	2.196	%	0.599	0.911							
		MSH6	2.293	%	0.521	0.965							
		PMS2	1.804	%	NA	0.720							
Linoleic acid %	Fatty Acids rations	MLH1	31.367	%	3.824	504.752	1.162	0.332					
		MSH2	31.544	%	5.381	456.042							
		MSH6	31.761	%	4.103	542.068							
		PMS2	30.731	%	NA	476.223							
Omega-3 %	Fatty Acids rations	MLH1	5.079	%	1.122	2.004	0.534	0.661					
		MSH2	4.660	%	1.697	1.951							
		MSH6	4.723	%	1.146	1.859							
		PMS2	4.400	%	NA	1.845							
Omega-6 %	Fatty Acids rations	MLH1	36.403	%	3.642	669.610	0.988	0.404					
		MSH2	36.451	%	5.791	640.818							
		MSH6	37.059	%	3.390	726.613							
		PMS2	35.868	%	NA	664.984							
PUFA %	Fatty Acids rations	MLH1	41.483	%	3.730	868.431	0.619	0.605					
		MSH2	41.110	%	5.699	830.225							
		MSH6	41.782	%	3.469	915.351							
		PMS2	40.268	%	NA	841.337							
MUFA %	Fatty Acids rations	MLH1	25.002	%	2.795	1.216	0.336	0.799					
		MSH2	25.789	%	4.803	1.217							
		MSH6	25.284	%	2.190	1.216							
		PMS2	28.562	%	NA	1.225							
SFA %	Fatty Acids rations	MLH1	33.515	%	1.823	0.500	1.664	0.184					
		MSH2	33.101	%	2.215	0.500							
		MSH6	32.934	%	1.635	0.500							
		PMS2	31.170	%	NA	0.499							
Glucose	Glycolysis related met	MLH1	5.894	mmol/l	0.970	0.876	2.950	0.06					
		MSH2	5.526	mmol/l	1.209	0.851							
		MSH6	6.192	mmol/l	1.184	0.879							
		PMS2	8.202	mmol/l	NA	0.936							
Pyruvate	Glycolysis related met	MLH1	0.073	mmol/l	0.035	-2.657	0.330	0.803					
		MSH2	0.061	mmol/l	0.021	-2.703							
		MSH6	0.079	mmol/l	0.036	-2.577							
		PMS2	0.078	mmol/l	NA	-2.422							
Citrate	Glycolysis related met	MLH1	0.068	mmol/l	0.009	-5.106	0.783	0.508					
		MSH2	0.068	mmol/l	0.008	-4.988							
		MSH6	0.071	mmol/l	0.011	-4.925							
		PMS2	0.065	mmol/l	NA	-4.967							
Acetate	Ketone bodies	MLH1	0.024	mmol/l	0.031	-14.769	4.535	0.014					
		MSH2	0.068	mmol/l	0.154	-12.225			0.281				
		MSH6	0.014	mmol/l	0.004	-17.779			0.103	0.013			
		PMS2	0.009	mmol/l	NA	-21.966							
Acetoacetate	Ketone bodies	MLH1	0.064	mmol/l	0.054	-2.836	1.874	0.143					
		MSH2	0.075	mmol/l	0.044	-2.466							
		MSH6	0.095	mmol/l	0.081	-2.399							
		PMS2	0.020	mmol/l	NA	-3.262							
Acetone	Ketone bodies	MLH1	0.027	mmol/l	0.017	-14.612	1.017	0.391					
		MSH2	0.030	mmol/l	0.012	-13.448							
		MSH6	0.034	mmol/l	0.023	-12.335							
		PMS2	0.017	mmol/l	NA	-16.398							
3-hydroxybutyrate	Ketone bodies	MLH1	0.127	mmol/l	0.124	-2.415	2.312	0.085					
		MSH2	0.176	mmol/l	0.113	-1.843							
		MSH6	0.259	mmol/l	0.283	-1.686							
		PMS2	0.054	mmol/l	NA	-2.462							
Albumin	Miscellaneous	MLH1	42.662	g/l	3.911	925.572	7.963	0					
		MSH2	38.987	g/l	5.202	725.605			**0**				
		MSH6	41.315	g/l	3.032	859.792			0.405	0.096			
		PMS2	38.285	g/l	NA	623.662							
Creatinine	Miscellaneous	MLH1	79.433	umol/l	14.648	2.418					Unequal variance p= 0.026	0.771	0.68
		MSH2	87.886	umol/l	24.138	2.426							
		MSH6	81.602	umol/l	20.879	2.416							
		PMS2	78.958	umol/l	NA	2.394							
Glycoprotein acetyls	Miscellaneous	MLH1	0.890	mmol/l	0.137	-0.111					Unequal variance p= 0.010	3.059	0.217
		MSH2	0.868	mmol/l	0.215	-0.160							
		MSH6	0.935	mmol/l	0.128	-0.114							
		PMS2	0.806	mmol/l	NA	-0.254							
Total cholines	Phospholipids	MLH1	3.115	mmol/l	0.438	1.556	4.766	**0.012**					
		MSH2	2.845	mmol/l	0.332	1.415			0.248				
		MSH6	2.831	mmol/l	0.317	1.343			**0.017**	**0.855**			
		PMS2	2.267	mmol/l	NA	1.034							
Phosphatidylcholines	Phospholipids	MLH1	2.632	mmol/l	0.406	1.112	4.914	**0.01**					
		MSH2	2.414	mmol/l	0.328	1.022			0.439				
		MSH6	2.342	mmol/l	0.288	0.931			**0.01**	0.592			
		PMS2	1.856	mmol/l	NA	0.693							
Phosphoglycerides	Phospholipids	MLH1	2.805	mmol/l	0.430	1.280	4.434	**0.016**					
		MSH2	2.561	mmol/l	0.363	1.168			0.38				
		MSH6	2.529	mmol/l	0.315	1.085			**0.019**	**0.747**			
		PMS2	2.009	mmol/l	NA	0.816							
Sphingomyelins	Phospholipids	MLH1	0.526	mmol/l	0.075	-0.493	4.420	0.016					
		MSH2	0.474	mmol/l	0.082	-0.543			0.22				
		MSH6	0.484	mmol/l	0.053	-0.561			0.028	0.932			
		PMS2	0.373	mmol/l	NA	-0.676							
TG/PG ration	Phospholipids	MLH1	0.552	ratio	0.258	-0.772	0.614	0.609					
		MSH2	0.624	ratio	0.479	-0.780							
		MSH6	0.518	ratio	0.182	-0.931							
		PMS2	0.816	ratio	NA	-0.407							
